# Comprehensive Integrative Analysis Reveals the Association of *KLF4* with Macrophage Infiltration and Polarization in Lung Cancer Microenvironment

**DOI:** 10.3390/cells10082091

**Published:** 2021-08-14

**Authors:** Shweta Arora, Prithvi Singh, Shaniya Ahmad, Tanveer Ahmad, Ravins Dohare, Saleh A. Almatroodi, Faris Alrumaihi, Arshad Husain Rahmani, Mansoor Ali Syed

**Affiliations:** 1 Translational Research Lab, Department of Biotechnology, Faculty of Natural Sciences, Jamia Millia Islamia, New Delhi 110025, India; shweta169213@st.jmi.ac.in (S.A.); shaniya169053@st.jmi.ac.in (S.A.); 2 Centre for Interdisciplinary Research in Basic Sciences, Srinivasa Ramanujan Block, Jamia Millia Islamia, New Delhi 110025, India; prithvi.mastermind@gmail.com (P.S.); ravinsdohare@gmail.com (R.D.); 3 Multidisciplinary Centre for Advance Research and Studies, Jamia Millia Islamia, New Delhi 110025, India; tahmad7@jmi.ac.in; 4 Department of Medical Laboratories, College of Applied Medical Sciences, Qassim University, Buraydah 51452, Saudi Arabia; smtrody@qu.edu.sa (S.A.A.); f_alrumaihi@qu.edu.sa (F.A.); ah.rahmani@qu.edu.sa (A.H.R.)

**Keywords:** *KLF4*, tumor-associated macrophages (TAMs), tumor microenvironment (TME), non-small cell lung cancer (NSCLC), macrophage polarization, *IL-1β*, miR-34a-5p

## Abstract

Macrophage polarization and infiltration to the tumor microenvironment (TME) is a critical determining factor for tumor progression. Macrophages are polarized into two states—M1 (pro-inflammatory, anti-tumorigenic and stimulated by LPS or *IFN-γ*) and M2 (anti-inflammatory pro-tumorigenic and stimulated by *IL-4*) phenotypes. Specifically, M2 macrophages enhance tumor cell growth and survival. Recent evidences suggest the pivotal role of microRNAs in macrophage polarization during the development of Non-small cell lung cancer (NSCLC), thus proposing a new therapeutic option to target lung cancer. In silico analysis determined cogent upregulation of *KLF4*, downregulation of *IL-1β* and miR-34a-5p in NSCLC tissues, consequently worsening the overall survival of NSCLC patients. We observed a significant association of *KLF4* with macrophage infiltration and polarization in NSCLC. We found that *KLF4* is critically implicated in M2 polarization of macrophages, which, in turn, promotes tumorigenesis. *KLF4* expression correlated with miR-34a-5p and *IL-1β* in a feed-forward loop (FFL), both of which are implicated in immune regulation. Mechanistic overexpression of miR-34a-5p in macrophages (*IL-4* stimulated) inhibits *KLF4*, along with downregulation of *ARG1*, *REL-1MB* (M2 macrophage specific markers), and upregulation of *IL-1β*, *IL-6*, (M1 macrophage specific markers), demonstrating macrophage polarization switch from M2 to M1 phenotype. Moreover, co-culture of these macrophages with NSCLC cells reduces their proliferation, wound healing, clonogenic capacity and enhanced NO-mediated apoptosis. Further, transfection of miR-34a-5p in NSCLC cells, also degrades *KLF4*, but enhances the expression of *KLF4* regulated genes—*IL-1β*, *IL-6* (pro-inflammatory mediators), which is further enhanced upon co-culture with *IL-4* stimulated macrophages. Additionally, we observed a significant increase in *i-NOS*/NO content upon co-culture, suggesting polarization reversion of macrophages from M2 to M1, and eventually leading to anti-tumor effects. Our findings thus show a significant role of *KLF4* in tumorigenesis and TAM polarization of NSCLC. However, miR-34a-5p mediated targeting of these molecular networks will provide a better therapeutic intervention for NSCLC.

## 1. Introduction

Lung cancer (LC) is one of the prime causes of cancer-associated mortality worldwide. It is divided into two types: (a) more fatal but less recurrent Small cell lung cancer (SCLC—16.8%), and (b) highly prevalent Non-small cell lung cancer (NSCLC—80.5%). NSCLC is again subdivided into large cell carcinoma (10%), lung squamous cell carcinoma (LUSC, 25%), and lung adenocarcinoma (LUAD, 40%). Besides the development of ingenious treatment strategies, the survival rate is still low (15%). This is attributed to tumor heterogeneity, poor prognosis, and the development of chemoresistance. Further, NSCLC therapy has attained saturation due to highly undulating gene expression profiles of tumors [1]. Over a short period of time, TME has emerged as one of the most callow and assuring fields in cancer research. Several studies have explored that immune microenvironment regulates both immunotherapy and prognosis of patients [2]. A report has identified the role of differences in immune cell composition in NSCLC microenvironment are correlated with survival and histopathological subtypes [3]. Another study has reported the role of the NSCLC immune microenvironment in prognosis after surgery [4]. Tumorigenesis is a chronic process and the cells surrounding tumors create a dynamic and conglomerate microenvironment that affects the progression and prognosis of tumors. Moreover, the functional state of immune cells in TME is one of the decisive mechanisms that govern tumor reversion [5]. Thus, understanding the immunophenotypes ramified in tumor immunity reactions and analyze the unique immune-related therapeutic targets in NSCLC becomes persuasive. 

Numerous recent studies have focused on various effects of tumor-associated macrophages (TAMs) and are found to be cynical for TME, by affecting several processes of tumorigenesis including proliferation, invasion, and metastasis of tumor cells. Macrophages are key multifunctional components of the immunological system that can be polarized into different phenotypic states—classically activated, pro-inflammatory M1-like TAMs, and alternatively activated immunosuppressive M2-like TAMs. M1/M2 polarity is dependent upon arginine metabolism via two opposing pathways. M1-like macrophages arise from the *iNOS* pathway, utilizing arginine to produce citrulline and NO, whereas M2-like macrophages originate from the arginase pathway, which utilizes arginine to produce urea and ornithine. Thus, *Arginase* and *iNOS* are critical components of M1/M2 macrophage polarization. M1 macrophages can be polarized by stimulation with lipopolysaccharides (LPS) and Th1 cytokines, including *IFN-γ* and *TNF-α*, and are characterized by *CD80*, *CD86*, *iNOS*, *TLR-2*, *TLR-4* and MHC-II surface phenotypes. They release a range of cytokines such as *IL-6*, *IL-1β*, *IL-12*, *TNF-α*, *IL-1α*, *CXCL9*, and *CXCL10*, which positively regulates the unpolarized macrophages and attracts them to acquire M1 macrophages. Transcription factors (TFs)—*NF-κβ*, *IRF3*, *IRF5*, *STAT1*, and *STAT5* regulate the expression of M1 genes. *NF-κβ* and *STAT1* are the dominant pathways involved in M1 macrophage polarization, leading to microbicidal and tumoricidal functions. On the other hand, M2 polarization occurs due to stimulation by *IL-4*, *IL-13*, *IL-33*, *IL-10*, and *TGF-β*. However, M2 macrophage activation is directly induced by *IL-4* and *IL-13*, and, cytokines such as *IL-33* and *IL-25* amplify M2 macrophage polarization, upon production of Th2 cytokines. They are characterized by the expression of surface markers, including *CD206*, *CD209*, *CD163*, *YM1/2* and *FIZZ1*. The cytokines—*CCL1*, *CCL17*, *CCL18*, *CCL22*, *CCL24*, *IL-10* and *TGF-β*, upon upregulation causes M2 polarization of unpolarized macrophages. *STAT6*, *JMJD3*, *IRF4*, *PPARγ* and *PPARδ* are the key TFs involved in M2 regulation. Moreover, *STAT6* is the predominant pathway involved in M2 activation. M2 macrophages are further subdivided into M2a, M2b, M2c and M2d, among which M2d macrophages promote angiogenesis and tumorigenesis, releasing *IL-10*. The expression profiles of cytokines and chemokines determine the depth of immune responses and inflammatory reactions [6,7,8,9,10]. Thus, both the phenotypes represent the extremities of TAM function, with M2 phenotype, being immensely associated with initiation, development, progression, and poor prognosis of cancers [11,12,13]. Noticeably, the cytokines present in TME allow TAMs to attain a pro-tumorigenic phenotype (M2 macrophage) and exert an immunosuppressive effect [14,15,16,17,18]. Overall, TAMs are substantially plastic cells, and their phenotypic reprogramming to extort an antitumor response is the focus of current research. 

*KLF4* is a highly conserved nuclear TF, containing a zinc-finger like domain, bearing N-terminal transcriptional activation domain, C-terminal transcription inhibitory region along with nuclear localization sequences for the regulation of protein interactions [19]. It is critically intricated into several aspects of cellular processes, including proliferation, differentiation, somatic reprogramming, tissue homeostasis and apoptosis [20]. Reports have identified its role in induction of fibroblasts into pluripotent stem cells, and designated it as the first gene regulating the transcription of *C-myc*, *Oct4*, and *Sox2* resulting in transformation of non-neoplastic epithelial cells to cancer stem cells [21,22,23]. It is found to express in both nucleus and cytoplasm; however, the subcellular localization affects the formation of cancer stem cells, thereby affecting drug resistance too [24]. The diversity of biological roles of *KLF4* can be attributed to multilevel regulation at transcription, miRNA, alternative splicing, post-translational modification (PTM), protein stability, protein interactions and subcellular localization levels. It is a bifunctional TF that may either activate or repress transcription, depending upon the target gene. Thus, cell type and context may determine the tumor suppressive and oncogenic role of *KLF4*. It is implicated in several roles in cancer; however, nearly all of the studies have suggested its tumor suppressive action, e.g., in gastric, colon and colorectal cancers, esophageal, lung and bladder cancer, T-cell leukemia and medulloblastoma [20,25,26,27,28,29,30,31]. Contrarily, it is overexpressed in head and neck, breast and skin cancer and acts as a transforming oncogene [32,33]. Moreover, tumor subtypes and microenvironment also play a convincing role. Further, its overexpression is associated with poor prognosis in several cancers [34]. Studies have identified its correlation with the clinicopathological features of patients with LC and is considered to be a probable biomarker of LC [20,35]. A recent study has identified a differential expression of *KLF4* in LC subtypes, with a significant downregulation in NSCLC and upregulation in SCLC. In addition, a compelling overexpression was also observed in advanced stages of NSCLC [20]. Furthermore, it is a demanding regulator of macrophage polarization. It has been found to be highly induced in *STAT6* regulated M2 macrophages and reduced in M1 macrophages via reclusion of coactivators needed for *NF-κβ* activation. Moreover, *KLF4* deficient macrophages display increased expression of pro-inflammatory genes, bactericidal effects and altered metabolism [36].

In this study, we have comprehensively analyzed the messenger RNA (mRNA) and microRNA (miRNA) transcriptome of NSCLC patients obtained from National Center for Biotechnology Information (NCBI)-Gene Expression Omnibus (GEO) datasets and their correlation with the prognosis of cancer patients using the Kaplan–Meier (KM) plotter database. We performed the pathway enrichment analysis to identify the immune related pathways in which differentially expressed genes (DEGs) were functionally enriched. Further, we analyzed the interactions between DEGs and differentially expressed miRNAs (DEMs) to identify the significant interacting Feed-Forward Loop (FFL) among them. We validated the expression levels using UALCAN and cBioportal databases. Further, we explored the infiltration of immune cells using Tumor Immune Estimation Resource (TIMER) database. We also performed in vitro co-culture of macrophages with NSCLC cells to validate the significant associations with components of FFL and macrophage polarization. The findings of this study have shed light on the considerable role of *KLF4* in infiltration and polarization of TAMs. Further, the inhibition of *KLF4* by miR-34a-5p leads to consequent inhibition of M2 markers and promotion of M1 markers. This has enabled us to identify the probable strategy of reversing the polarization of TAMs. More strikingly, reversal of TAM polarization promotes antitumor activities in NSCLC.

## 2. Materials and Methods

### 2.1. Identification of NSCLC-Specific DEGs, DEMs and Pathway Enrichment Analysis

The miRNA and mRNA expression profiles of NSCLC patients were downloaded from the NCBI-GEO (https://www.ncbi.nlm.nih.gov/geo/, accessed on 15 March 2021) database [37]. GEO was queried utilizing ‘Non-small cell lung cancer’ and ‘NSCLC’ as appropriate keywords. The search results were further trimmed down by applying following inclusion criteria: (1) the datasets must be obtained from non-tumor and adjacent tumor tissues of NSCLC patients; (2) the datasets must be standardized or raw; (3) the datasets must be miRNA/mRNA transcriptome data of the whole genome; (4) the datasets should be “expression profiling by array” type and its samples must belong to “Homo Sapiens”; and (5) the datasets must have more than 100 samples. GEO2R (https://www.ncbi.nlm.nih.gov/geo/geo2r/, accessed on 15 March 2021) was utilized to detect the DEGs and DEMs between NSCLC and normal controls expression profiles. The probe IDs with their corresponding Hugo Gene Nomenclature Committee (HGNC) gene symbols as NA were discarded. Duplicate gene symbols corresponding to multiple probe IDs were removed by averaging their relative expression values. The adjusted *p*-values (adj.*p*) were computed using the Benjamini–Hochberg (BH) False Discovery Rate (FDR) method to correct for the likelihood of false-positive results [38]. DEGs were screened using an adj.*p* < 0.001 and $\left|log_{2}\left(fold\;change\right)\right|>2$ as the preferred threshold. Additionally, DEMs were screened using an adj.*p* < 0.0001. The up and downregulated DEGs were filtered considering adj.*p* < 0.0001 with $log_{2}\left(fold\;change\right)>2$ and adj.*p* < 0.0001 with $log_{2}\left(fold\;change\right)<-2$. Additionally, up, and downregulated DEMs were filtered considering adj.*p* < 0.0001 with $log_{2}\left(fold\;change\right)>0$ and adj.*p* < 0.0001 with $log_{2}\left(fold\;change\right)<0$. Reactome library available within the Enrichr database [39] was utilized for pathway enrichment analysis of DEGs where top 10 significant pathways corresponding to *p*-value < 0.05 were selected. 

### 2.2. Extraction of Significant NSCLC-Specific miRNAs and TFs

TF-gene regulation: TF-gene interaction pairs were extracted from the ChIPBase v2.3 database [40] and Enrichr platform TF libraries like ChEA, TRANSFAC and JASPAR PWMs. TFs with binding sites located within 1kb upstream region were retrieved from ChIPBase database, whereas the TFs with a significant *p*-value < 0.05 were retrieved from Enrichr TF libraries. The list of TFs were scrutinized via literature studies and only NSCLC-specific TFs were retained

miRNA-gene/TF repression: miRNA-gene/TF pairs were extracted from miRWalk v3.0 [41], miRSearch v3.0 [42] and Starbase v2.0 [43] databases, respectively. Parameters like binding gap = 1, 3′UTR region, and score > 0.95 were considered as the cut-off for significant miRNAs retrieval from miRWalk. miRNAs having a significantly high score and denoted by the green-colored band were extracted from miRSearch.

The list of miRNAs obtained from miRNA-gene/TF repression pairs overlapping with NSCLC-specific DEMs and literature-validated NSCLC-specific miRNAs were retained. Finally, all the three types of regulatory interaction pairs were altered with respect to these finalized NSCLC-specific TFs and miRNAs. All these molecular interactions were then merged to construct a NSCLC-specific 3-node miRNA FFL [44] and subsequently visualized using Cytoscape v3.8.2 [45].

### 2.3. Overall Survival (OS) Analysis 

The KM plotter database (https://kmplot.com/analysis/, accessed on 31 March 2021) [46] was used to evaluate the prognostic value of *KLF4*, *IL-1β* and miR-34a-5p in The Cancer Genome Atlas (TCGA)-LUAD cohort. Clinical data from 513 LUAD patients (mRNA-Seq and miRNA-Seq) including smoking history, pathological grades, histology, clinical staging, and gender were retrieved from the KM plotter. The cohorts of patients were divided by median expression values via auto-select best cut-off. In accordance with the median expression level of these genes, the LUAD patient samples were bifurcated into two groups, i.e., high, and low expression groups. Then, the log-rank *p*-values, number at risk, hazard ratio (HR), and 95% confidence interval (CI) were computed. *p*-value < 0.01 was considered as the statistically significant threshold.

### 2.4. Validation of KLF4 and IL-1β Using UALCAN and cBioPortal

UALCAN (http://ualcan.path.uab.edu/, accessed on 3 April 2021) [47] and cBioPortal (https://www.cbioportal.org/, accessed on 3 April 2021) [48,49] web-based tools were queried in order to validate the roles of *KLF4* and *IL-1β* in TCGA-LUSC and Pan-lung cancer cohorts, respectively. Validation of expression levels of *KLF4* and *IL-1β* was done using UALCAN with respect to clinical characteristics such as nodal metastasis and TP53 mutation status in LUSC. Graphical summarization of genomic alterations including mutations and CNA (amplifications and homozygous deletions) within non-small cell lung cancer dataset (TCGA, Pan Cancer Atlas) was presented using cBioPortal. 

### 2.5. Tumor Immune Infiltration Analysis

TIMER web-based tool (http://timer.cistrome.org/, accessed on 3 April 2021) [50] was queried to explore the interconnection between tumor-infiltrating immune cells in TCGA-LUAD and LUSC patients and expression levels of *KLF4* and *IL-1β*. Spearman’s test was used and *p*-value < 0.05 was regarded as the statistically significant threshold.

### 2.6. Cell Culture, Macrophage Differentiation, and Transient Transfection

A549, H1299 (Human NSCLC cell lines), and THP-1(Human monocytes) were obtained from NCCS, Pune, India and maintained in RPMI1640 (Cat # 61870036, Gibco, Waltham, MA, USA), 1% antibiotic-antimycotic (Cat # 15240096, Gibco, Waltham, MA, USA) and incubated at 37 °C and an atmosphere of 5% CO_2_. THP-1 cells were differentiated using 5 ng/mL PMA (P8139, Sigma, Saint Louis, MO, USA) and stimulated using *IL-4* (10 ng/mL, Cat # I4269, Sigma Aldrich, Bangalore, India) for 24 h. THP-1 and H1299 cells were transiently transfected with 30 pmol/mL miR-34a-5p mimic (Qiagen, Hilden, Germany) and scrambled miRNA mimic (Qiagen, Hilden, Germany) using lipofectamine 3000 (Invitrogen, Waltham, MA, USA) for 48 h. Overexpression plasmid, i.e., pcDNA3.1 containing the full-length sequence of *KLF4*, and empty plasmid (Addgene, Watertown, MA, USA) were also transfected at a concentration of 900 ng/mL along with miR-34a mimic into THP-1, A549, and H1299 cells.

### 2.7. Co-Culture 

Transfected THP-1 cells were directly co-cultured with A549 cells in a density of 1: 10 for 24 h. On the other hand, H1299 cells (transiently transfected with miR-mimic and scrambled) were directly co-cultured with THP-1 cells (stimulated with 20 ng/mL *IL-4*) in a density of 1:10 for 24 h. Co-culture conditioned medium (CM) was collected, briefly centrifuged, and stored at −80 °C for further use. 

### 2.8. Quantitative Real-Time PCR (qRT-PCR)

Total RNA was isolated from transfected THP-1 and H1299 cells alone as well as when co-cultured using TRIzol reagent (Ambion, Austin, TX, USA) according to the manufacturer’s protocol. cDNA was then reverse transcribed using the iScript cDNA synthesis kit (Bio-Rad, Hercules, CA, USA). qRT-PCR analyses for mRNA of *KLF4*, *REL-1MB*, *IL-6*, *IL-1β*, and *GAPDH* were performed by using iTaq Universal SYBR Green Supermix (Bio-Rad, Hercules, CA, USA). PCR was performed using 7900HT Fast Real-time PCR System (Applied Biosystems, Waltham, MA, USA) using GAPDH as an endogenous control. Relative quantification from real-time data is presented, based on the calculation of 2^−ΔΔCt^. The primer sequences are available in Supplementary Material (Oligonucleotide sequences and list of antibodies used).

### 2.9. One-Step qRT-PCR

To confirm the transfection of miR-34a-5p, cDNA was synthesized in 5X miScript Hi-Spec Buffer using mi-Script (II) RT kit (Qiagen, Hilden, Germany) using manufacturers’ protocol. miR-34a-5p expression was measured by the miScript SYBR Green PCR Kit (Qiagen, Hilden, Germany) using manufacturers’ protocol. Relative expression was quantified using ΔΔCt method and RNU6 as a normalizing control.

### 2.10. Western Blotting

Cells were lysed using RIPA Lysis and Extraction Buffer (Thermo Scientific, Waltham, MA, USA) supplemented with protease and phosphatase inhibitor. Isolated proteins were estimated using Bradford Reagent (Bio-Rad, Hercules, CA, USA). Equal concentration of protein (20 µg) was separated via 12.5% sodium dodecyl sulphate–polyacrylamide gel electrophoresis (SDS—PAGE) and blotted onto polyvinylidene difluoride (PVDF) membrane (Millipore, USA). After blocking with 5% skimmed milk, blots were incubated in primary antibody (1:1000 dilution) overnight, followed by incubation with secondary antibody conjugated to HRP (1:1000 dilution). Protein bands were visualized on X-ray films using Clarity Max ECL Western Blotting Substrates (Bio-Rad, Hercules, CA, USA). 

### 2.11. Flow Cytometry

Measurement of apoptosis was done in A549 and H1299 cells after treatment with CM for 48 h using Dead Cell Apoptosis Kit with AnnexinV-FITC and PI, for flow cytometry (Invitrogen, Waltham, MA, USA) using the manufacturers’ protocol. Fluorescence emission was measured by BD FACS Aria™ III (BD Biosciences, Franklin Lakes, NJ, USA) using BD FACSDiva software.

### 2.12. Cell Viability and Cell Proliferation

A549 and H1299 cells were treated with CM for 48 h, and the cell viability was determined using 0.5% Trypan blue staining. Cell proliferation was determined using MTT assay (Cat #634844, Merck, Bangalore, India). Briefly, 7500 cells/well were seeded in a 96-well plate and treated with CM for 48 h, after which 0.5 mg/mL MTT was added for 3 h. DMSO was added, and the cells were incubated at room temperature for 30 min. Finally, absorbance was measured at 540 nm. 

### 2.13. Clonogenic Assay

A549 and H1299 cells were treated with CM for 48 h and then allowed to form colonies in a serum-deprived medium for 12 days. Colonies were stained with 0.5% crystal violet in methanol, counted, and solubilized in a destain solution of 10% acetic acid. OD of the extracts were spectrophotometrically determined at 570 nm.

### 2.14. In Vitro Scratch Assay

A549 and H1299 cells were treated with CM for 48 h and were allowed to grow as a monolayer after which a scratch was created using a sterile pipette tip. Scratched cells were removed by washing with PBS and allowed to migrate through the scratch for 48 h in a serum-deprived medium. The cells were photographed at different intervals of time and the area covered by scratch was measured using ImageJ software.

### 2.15. Nitrite Assay

The concentration of nitrites in CM was determined using Griess reagent (Chromous Biotech, Bangalore, India) using the manufacturers’ protocol. NaNO2 was used to prepare respective standards.

### 2.16. Dual-Luciferase Assay

p-MIR reporter plasmid containing 3′UTR of *KLF4* and plasmid containing the mutated sequence of *KLF4* were purchased from Addgene. Dual-luciferase assay was performed using the DLR assay kit (Cat # E1910, Promega, Madison, WI, USA) as per the manufacturer’s protocol using plasmid expressing the Renilla luciferase gene (pRL, Promega, Madison, USA, 20 ng) as a control. Luciferase activity was assayed on an InfiniteM200 Pro Multimode Reader (TECAN, Seestrasse, Männedorf, Switzerland). Relative fold change in luciferase activity was estimated, following normalization to Renilla luciferase activity.

### 2.17. Statistical Analysis

All experiments were performed thrice independently, and the data is presented as mean ± SEM. Student’s *t*-test, One-Way Analysis of Variance (ANOVA) and Two-Way ANOVA were used to analyze the statistical significance of data. *p*-value < 0.05 was considered to be statistically significant. Statistical calculations were performed using Graph Pad Prism 7.0. 

## 3. Results

### 3.1. Identification of NSCLC-Specific DEGs, DEMs and Pathway Enrichment Analysis

**Figure 1:**
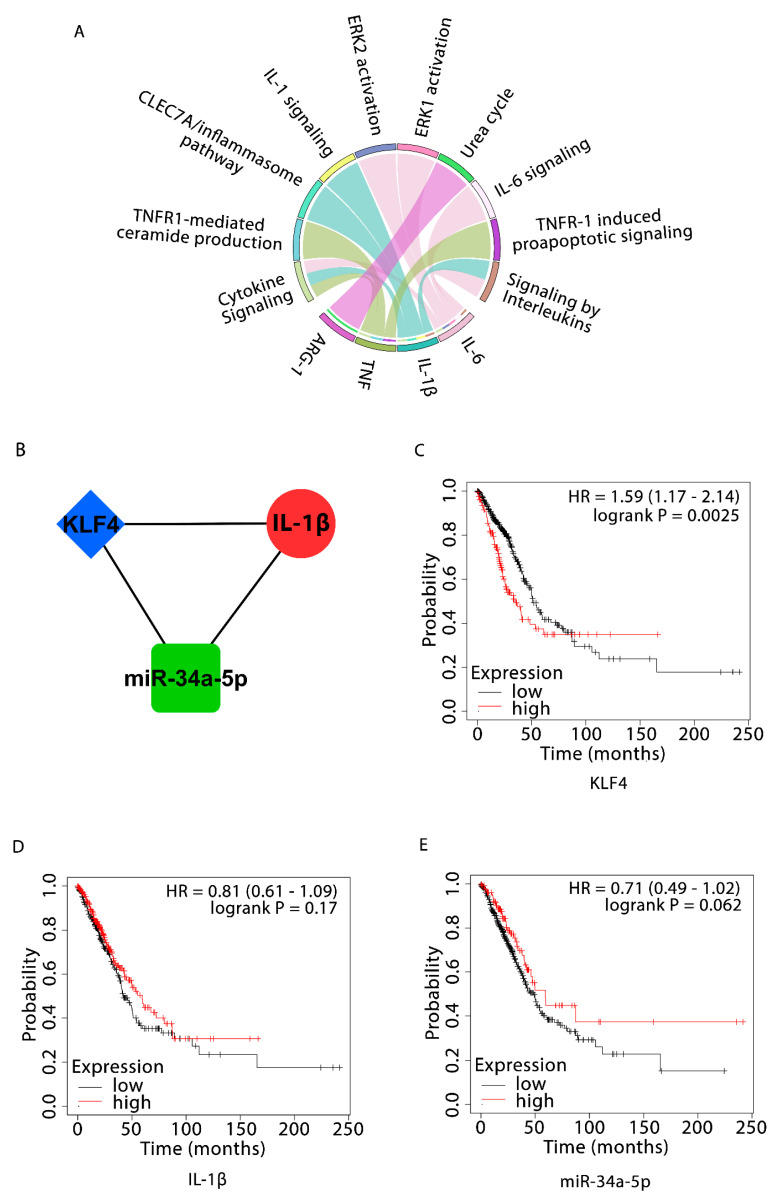
Identification of NSCLC-specific DEGs, DEMs, pathway enrichment analysis, 3-node miRNA FFL and OS analyses. (**A**) Chord plot showing the association of top 10 significant pathways and 4 DEGs involved in them. The outer circle indicates top 10 significant pathways (on the top semicircle) and 4 DEGs (on the bottom semicircle). Each DEG has a unique colored band and colored edge inside the circle represents relationship of a particular DEG with respective connected pathway(s); (**B**) Higher-order network motif consisting of one miRNA (miR-34a-5p) as green rectangular node, one gene (*IL-1β*) as *red circular node*, and one TF (*KLF4*) as *blue diamond node* along with their relative regulatory interactions. Kaplan–Meier survival curves showing the prognostic values of (**C**) *KLF4*; (**D**) *IL-1β*; and (**E**) miR-34a-5p expression levels across TCGA-LUAD cohort. The high and low expression curves are signified by red and black colors, respectively. HR = Hazard Ratio.

Based on the specified searching and inclusion criteria, we chose NSCLC-specific mRNA and miRNA expression profiles possessing accession numbers GSE75037 and GSE53882. In consideration with the abovementioned threshold, a total of 612 DEGs and 467 DEMs were identified from datasets GSE75037 and GSE53882, respectively. Amongst these, a total of 199 and 413 DEGs along with 247 and 220 DEMs were categorized as up and downregulated (Supplementary Tables S1 and S2). Chord plot representing the association of top 10 significant pathways with 4 participating DEGs (i.e., *ARG1*, *TNF*, *IL-6*, *IL-1β*) is shown in Figure 1A. Clearly, the interaction edges in the plot show that IL-6 was present in maximum number of pathways (i.e., 5). All four pathway enriched DEGs were used for further analysis. 

### 3.2. NSCLC-Specific 3-Node miRNA FFL and *OS Analyses*

The NSCLC-specific 3-node miRNA FFL comprised 49 nodes, among which four were pathway enriched DEGs, 22 were NSCLC-specific miRNAs, and 23 were NSCLC-specific human TFs; and 242 edges, among which 22 belonged to miRNA-gene pairs, 37 to TF-gene pairs, and 183 to miRNA-TF pairs (Supplementary Figure S1, Table 1). Only a few nodes had a higher degree, with maximum nodes having a low degree for miRNAs, TFs, and genes in the miRNA-FFL, as shown by the right skewing of the node degree distribution (Supplementary Figure S2). One of the most strongly connected subnetwork motif (based on the degree) inside FFL included one TF (*KLF4*), one gene (*IL-1β*), and one miRNA (miR-34a-5p) (Figure 1B). The KM plot analysis of *KLF4* showed that higher expression levels of *KLF4* (HR = 1.59; 95% CI = 1.17–2.14; *p*-value < 0.05) worsened OS (Figure 1C), but higher expression of *IL-1β* (HR = 0.81; 95% CI = 0.61–1.09; *p*-value >0.05, nonsignificant) (Figure 1D) and miR-34a-5p (HR = 0.71; 95% CI = 0.49–1.02; *p*-value < 0.05) (Figure 1E) improved OS in patients with LUAD (Table 2). 

### 3.3. Validation of KLF4 and IL-1β Using UALCAN and cBioPortal

**Figure 2:**
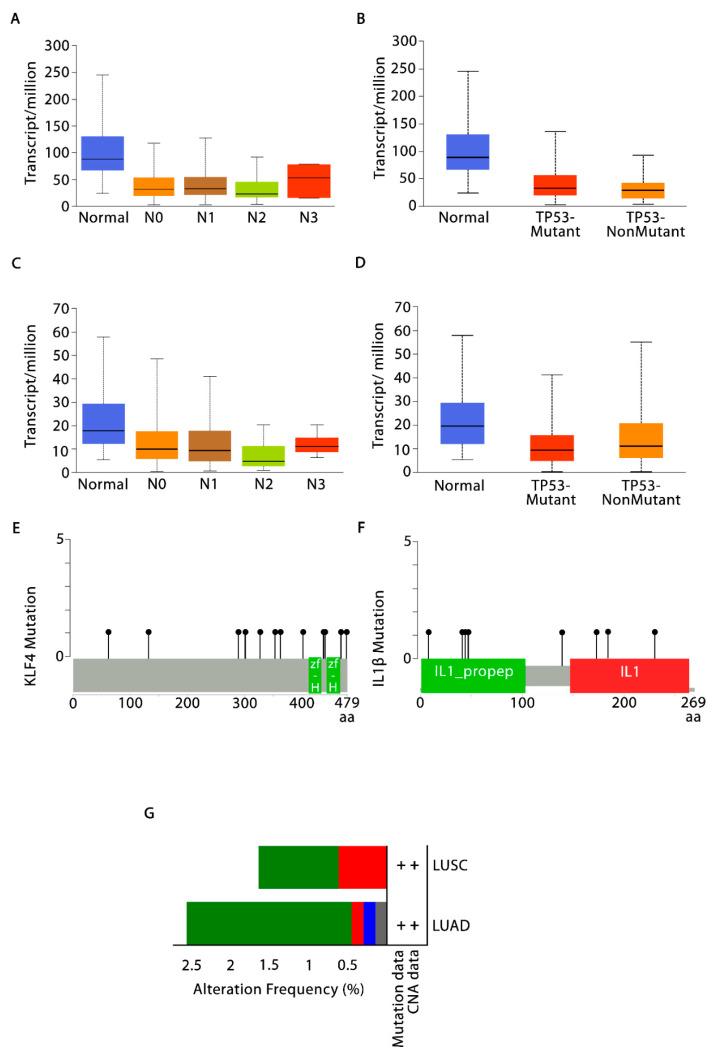
Validation of *KLF4* and *IL-1β* using UALCAN and cBioPortal. Box-and-whisker plots displaying *KLF4* expression levels in TCGA-LUSC cohort based on (**A**) nodal metastasis statuses; and (**B**) TP53 mutation via UALCAN database; (**C**) box-and-whisker plots showing levels of *IL-1β* expression in TCGA-LUSC cohort based on nodal metastasis statuses; and (**D**) TP53 mutation. Lollipop plots displaying 12 and 8 nonsynonymous mutations in (**E**) *KLF4* and (**F**) *IL-1β* protein domains. The grey-colored horizontal bar represents the whole length of *KLF4* and *IL-1β* proteins with the number of amino acids being displayed below the grey bar. Protein domains were showed by the red-and green-colored boxes. The locations and frequencies of mutations were denoted by the solid vertical lines and lollipop-like dots at their ends, respectively. (**G**) Barplots showing the alteration frequency of *KLF4* and *IL-1β* across TCGA-NSCLC cohort (2.58% of 660 LUAD cases and 1.65% of 484 LUSC cases). Green, red, blue, and grey colored bars depicts missense mutations (2.12% in LUAD and 1.03% in LUSC), amplification (0.15% in LUAD and 0.62% in LUSC), deep deletion (0.15% in LUAD), and truncating mutation (0.15% in LUAD), respectively.

UALCAN database was used to validate the *KLF4* and *IL-1β* expression in TCGA-LUSC cohort based on various clinicopathological features (i.e., nodal metastasis and TP53 mutation status). As shown in Figure 2A, the expression levels of *KLF4* significantly correlated with nodal metastasis [Normal vs. N0, Normal vs. N1, Normal vs. N2, Normal vs. N3] and TP53 mutation status in LUSC cohort (Figure 2B). Additionally, the expression levels of *IL-1β* significantly correlated with nodal metastasis [N0 vs. N2] (Figure 2C) and non-significantly with TP53 mutation status in LUSC cohort (Figure 2D). cBioPortal was used to investigate the specific genetic alterations of *KLF4* and *IL-1β* in NSCLC dataset (TCGA, Pan Cancer Atlas) with 1144 patient samples (660 LUAD + 484 LUSC). The Lollipop plot as shown in Figure 2E displayed the frequency and location of all possible mutations in Pfam protein domains. *KLF4* had a somatic mutation frequency of 1.0% (i.e., 12 missense mutations) and *IL-1β* had a somatic mutation frequency of 0.7% (i.e., 7 missense mutations + 1 truncating mutation), suggesting the significance of somatic mutations in their functional alterations (Figure 2F). Figure 2G showed the alteration frequency barplot of *KLF4* and *IL-1β* with missense mutation frequency of 2.12% (14 samples), deep deletion frequency of 0.15% (1 sample), amplification frequency of 0.15% (1 sample), and multiple alterations/truncating mutation frequency of 0.15% (1 sample) in case of LUAD. Whereas in LUSC, a missense mutation frequency of 1.03% (5 samples) and amplification frequency of 0.62% (3 samples) was observed. 

### 3.4. Evaluation of Tumor Immune Cell Infiltration

**Figure 3:**
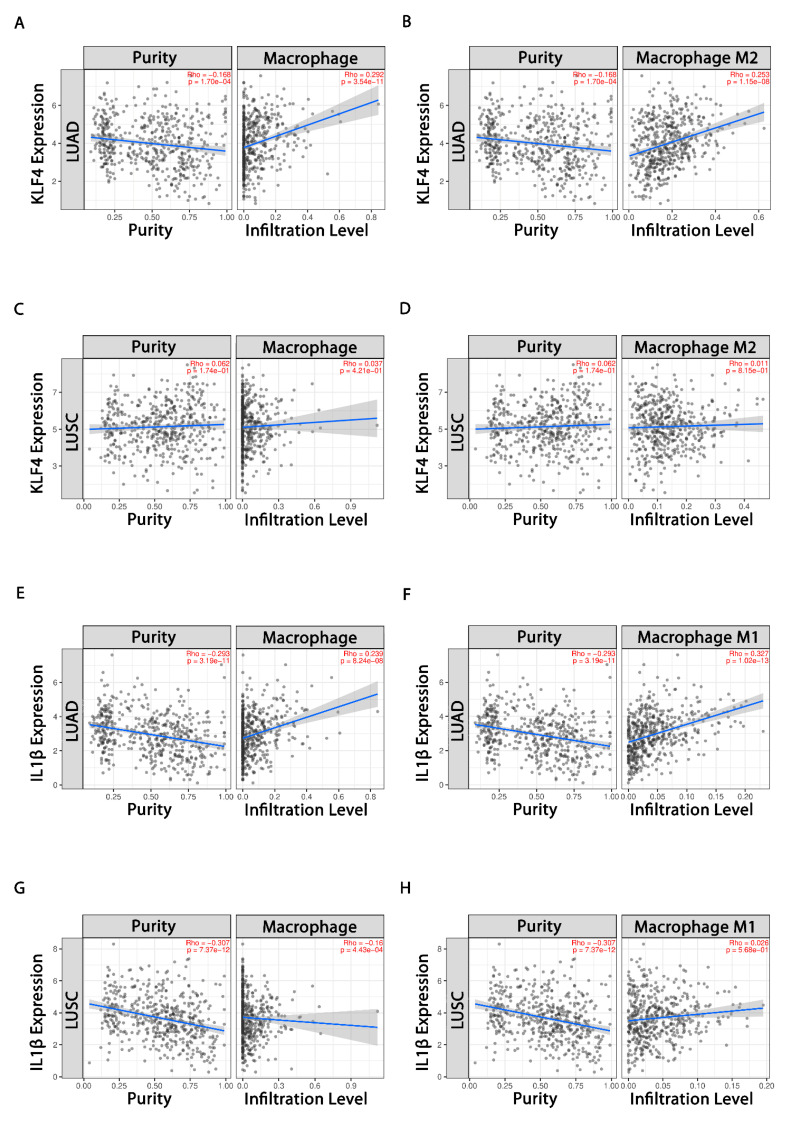
Tumor immune infiltration analysis. (**A**,**B**) Scatter plots exhibiting correlations of *KLF4* with infiltrating levels of TAMs and M2 macrophages in LUAD, respectively; (**C**,**D**) scatter plots showing correlations of *KLF4* with infiltrating levels of TAMs and M2 macrophages in LUSC, respectively; (**E**,**F**) scatter plots showing correlations of *IL-1β* with infiltrating levels of TAMs and M1 macrophages in LUAD; (**G**,**H**) scatter plots showing correlations of *IL-1β* with infiltrating levels of TAMs and M1 macrophages in LUSC. The left and right panel demonstrate gene expression levels against tumor purity and infiltrating levels of immune cells, respectively. In addition, Spearman’s correlation value and estimated statistical significance are shown as the legends for each scatter plot.

For assessing the significant correlation of *KLF4* and *IL-1β* with tumor purity and tumor-infiltrating functional immune cells in LUAD and LUSC, TIMER database was queried. The expression level of *KLF4* showed significant positive correlations with infiltrating levels of macrophages (r = 0.292, *p* = 3.54 × 10^−11^) (Figure 3A) and M2 macrophages (r = 0.253, *p* = 1.15 × 10^−8^) (Figure 3B) in LUAD. It also showed nonsignificant positive correlations with infiltration of macrophages (r = 0.037, *p* = 4.21 × 10^−1^) (Figure 3C) and M2 macrophages (r = 0.011, *p* = 8.15 × 10^−1^) (Figure 3D) in LUSC. *KLF4* expression also displayed significant negative correlations with tumor purity in LUAD (r = −0.168, *p* = 1.70 × 10^−4^), and nonsignificant positive correlations with tumor purity in LUSC (r = 0.062, *p* = 1.74 × 10^−1^), respectively. Additionally, *KLF4* showed significant negative correlations with infiltrating levels of NK cell (r = −0.18, *p* = 5.6 × 10^−5^), and B-Cell (r = −0.157, *p* = 4.54 × 10^−4^) in LUAD, whereas *KLF4* showed significant negative correlations with infiltrating levels of M1 macrophage (r = −0.085, *p* = 6.45 × 10^−2^) and NK cell (r = −0.132, *p* = 3.78 × 10^−3^); and nonsignificant negative and positive correlations with B Cell (r = −0.064, *p* = 1.65 × 10^−1^) and T Cell CD4+ (r = 0.043, *p* = 3.43 × 10^−1^) in LUSC (Supplementary Figure S3). Moreover, *IL-1β* shared positive correlation with macrophages (r = 0.239, *p* = 8.24 × 10^−8^) (Figure 3E) and M1 macrophage (r = 0.327, *p* = 1.02 × 10^−13^) (Figure 3F) in LUAD. Contrarily, it displayed negative correlation with macrophages (r = −0.16, *p* = 4.43 × 10^−4^) (Figure 3G), but nonsignificant positive correlation with M1 macrophages (r = 0.026, *p* = 5.68 × 10^−1^) (Figure 3H) in LUSC. Moreover, it also shared nonsignificant and significant negative correlations with B cells in LUAD (r = −0.035, *p* = 4.34 × 10^−1^) and LUSC (r = −0.199, *p* = 1.15 × 10^−5^), respectively (Supplementary Figure S3). The above findings highlight the significance of *KLF4* in enhanced infiltration of macrophages, specifically M2 subtypes. However, *IL-1β* favors infiltration of M1 macrophages.

### 3.5. KLF4, IL-1β and miR-34a-5p Is Associated with Macrophage Polarization

**Figure 4:**
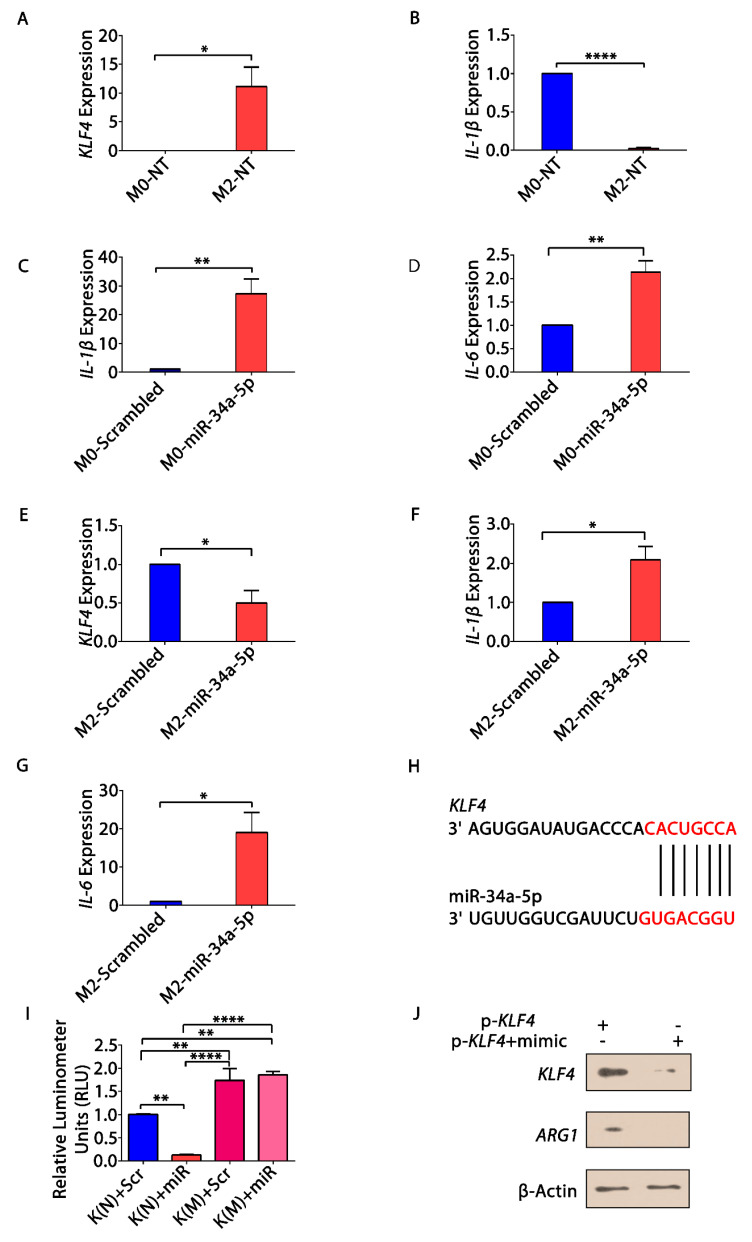
Association of *KLF4*, *IL-1β* and miR-34a-5p in macrophage polarization. Relative fold change in expression of (**A**) *KLF4* and (**B**) *IL-1β* in M0 (unstimulated) and M2 (IL4 stimulated) macrophages, as determined by qRT-PCR; relative fold change in expression of (**C**) *IL-1β* and (**D**) *IL-6 *upon transfection of miR-34a-5p in M0 macrophages; relative fold change in expression of (**E**) *KLF4*; (**F**) *IL-1β*; and (**G**) *IL-6* upon transfection of miR-34a-5p in M2 macrophages, determined by qRT-PCR; (**H**) position 25-32 region on *KLF4* 3′UTR showing conserved 8-mer binding site of miR-34a-5p (obtained from TargetScan); (**I**) dual-luciferase reporter assay—plasmid containing 3′UTR of KLF4 K(N) and mutated 3′ UTR of KLF4 K(M) were co-transfected with scrambled (Scr), miR-34a-5p mimic and pRL-vector containing Renilla luciferase gene, respectively, in A549 cells, and luminescence was measured. Measurements were expressed as relative luminometer units and normalized by the luminescence of Renilla luciferase; (**J**) THP-1 cells were transfected with *KLF4* overexpression plasmid and miR-mimic. Total proteins were extracted and evaluated for degradation of *KLF4* and *ARG1* by Western blotting. β-actin was used as an endogenous control. * *p* < 0.05, ** *p* < 0.01, **** *p* < 0.0001.

*KLF4* is known to be associated with M2 phenotype of macrophages [51]. Besides, *IL-1β* and *IL-6*, are also implicated in macrophage polarization [52], but their association with miR-34a-5p is not yet explored. For this, we analyzed the expression levels of *KLF4* and *IL-1β* in different phenotypic subtypes of macrophages—M0 (unstimulated) and M2 (stimulated by *IL-4*). Macrophages were actually divided in 3-subsets, M0, M2 as stated above along with M1 (LPS—stimulated). Macrophage stimulation was checked by semi-quantitative RT-PCR (Supplementary Figure S4). Further, we have used only M0 and M2 subsets in the study, as M2 macrophages are highly predominant in tumor microenvironment). qRT-PCR analysis revealed higher expression of *KLF4* (Figure 4A) and reduced expression of *IL-1β* (Figure 4B) in M2 subset as compared to M0 subset. To scrutinize the role of miR-34a-5p in macrophage polarization, the M0 and M2 subsets were transfected with scrambled and miR-34a-5p mimic. The relative expressions of M1 and M2 specific markers in macrophage subsets, were checked by semi quantitative RT-PCR (Supplementary Figures S5 and S6). We observed an increase in the expression of *IL-1β* (Figure 4C) and *IL-6* (Figure 4D) in M0 subset transfected with miR mimic. However, there was a decrease in *KLF4* (Figure 4E) and an increase in the expression of *IL-1β* (Figure 4F) and *IL-6* (Figure 4G), upon transfection of M2 subset with miR-mimic. These results demonstrated that *KLF4* is highly expressed in M2 subset and miR-34a-5p favors an increase in M1 pro-inflammatory markers (*IL-1β* and *IL-6)*. Furthermore, we found that *KLF4* is a direct target of miR-34a-5p, identified by TargetScan (Figure 4H). To confirm this, we have performed luciferase assay and found an eloquent decrease (>50%) in luciferase activity upon co-transfection of pMIR-*KLF4*—wild type 3′UTR (pMIR-*KLF4*-3′UTR), pRL vector, and miR-34a-5p mimic as compared to the mutated (co-transfection of pMIR-*KLF4*-3′UTR mutated, pRL vector, and scrambled) in A549 cells. In contrast, changes in luciferase activity upon co-transfection of miR-34a-5p mimic with the pMIR-*KLF4* mutant 3′UTR (pMIR-*KLF4*-m3′ UTR) were not so noteworthy, connotating the direct binding between miR-34a-5p and *KLF4* 3′UTR (Figure 4I). To assess whether or not the expression of *KLF4* protein is affected by miR-34a-5p transfection, we transfected pMIR-*KLF4*-3′UTR, and pMIR-*KLF4*-3′UTR in combination with miR-34a-5p mimic in unstimulated THP-1 cells. We analyzed the protein expression of transfected cells and found that miR-34a-5p reduced *KLF4* and *ARG1* (M2 specific marker) expression at the protein levels too (Figure 4J). These findings highlighted the significance of *KLF4* in polarization of TAMs towards M2 phenotype; however, miR-34a-5p directly targets *KLF4* and inhibits M2 phenotype, but promotes M1 phenotype, demonstrating their relevance in macrophage reprogramming.

### 3.6. KLF4—Acting as an Oncogene in NSCLC

**Figure 5:**
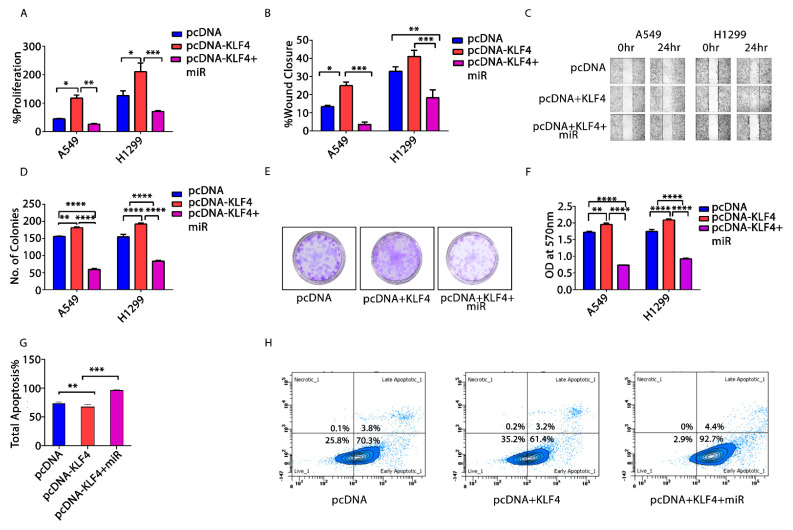
Oncogenic role of *KLF4* in NSCLC. *pcDNA*, *pcDNA-KLF4* and *pcDNA-KLF4* along with miR-34a-5p mimic were transfected, respectively, in A549 and H1299 cells and analyzed for cellular proliferation, wound healing, and clonogenic capacity after 48 h of treatment; (**A**). Cellular proliferation was checked via MTT assay; (**B**). In vitro scratch assay was performed to evaluate the migrative capacity of cells. % wound closure was recorded for all the groups in both A549 and H1299 cells till 24 h; (**C**). Pictures showing wound closure after 24 h in pcDNA, *pcDNA+KLF4*, and *pcDNA+KLF4+miR* transfected groups; (**D**). Clonogenic assay—number of colonies counted by ImageJ software for determination of clonogenic capacity of both A549 and H1299 cells upon CM treatment; (**E**). Pictures showing colonies appeared after treatment in H1299 cells; (**F**). OD at 570 nm was measured after destaining the colonies appeared after transfection; (**G**). Apoptotic analysis using AnnexinV–FITC staining of transfected A549 cells; (**H**). Fluorescence emission by transfected cells measured by flow cytometry. * *p* < 0.05, ** *p* < 0.01, *** *p* < 0.001, **** *p* < 0.0001. All the experiments were done thrice (N = 3).

Bioinformatics analysis has shown that *KLF4* is upregulated (Supplementary Table S1) in NSCLC cells. Thus, to explore the role of *KLF4* in NSCLC, we transfected A549 and H1299 cells, respectively, with empty pcDNA3.1, pcDNA3.1-*KLF4*-full length (FL), and pcDNA3.1-*KLF4* (FL) along with miR-34a-5p mimic. There was a significant increase in cellular proliferation, migration, and clonogenic capacity of NSCLC cells upon transfection of p*KLF4* (FL) (Figure 5A–F). Meanwhile, the percentage of apoptotic cells (A549) was significantly decreased with p*KLF4* (FL) transfection. However, all the above hallmarks were significantly decreased, and apoptosis increased upon co-transfection of pcDNA3.1-*KLF4* (FL) and miR-34a-5p mimic (Figure 5G,H). These results thus show that *KLF4* acts as an oncogene in the progression of NSCLC and its inhibition by miR-34a-5p counter these effects.

### 3.7. miR-34a-5p/KLF4 Mediated Macrophage Polarization Reduce Tumorigenesis

**Figure 6:**
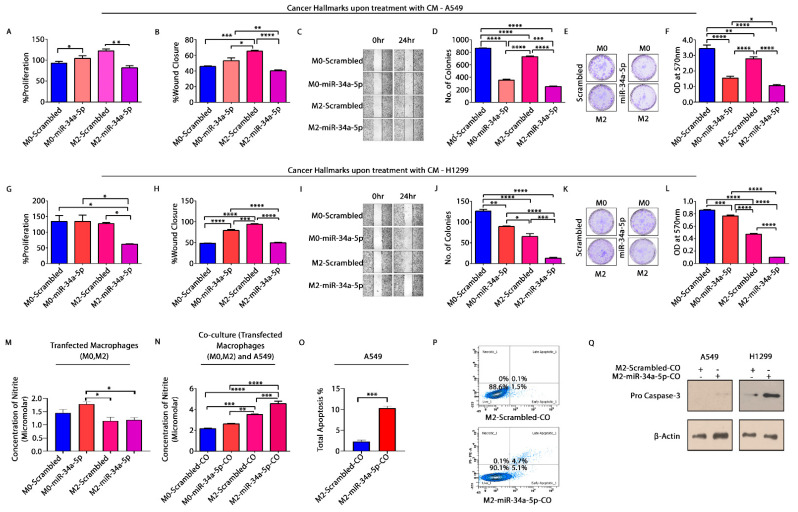
Macrophage polarization affects cancer hallmarks. Transfected M0 and M2 macrophages were co-cultured with A549 cells. CM was collected and used to check cancer hallmarks on A549 and H1299 cells. (**A**). Cellular proliferation—graph showing % proliferation of A549 cells, determined by MTT Assay; (**B**,**C**). % wound closure of the scratch created in monolayer and photographic images of scratches at different time intervals (0 h, 24 h); (**D**–**F**). Colony counts photographic images showing colonies and spectrophotometric determination of clonogenic capacity of A549 cells upon treatment with co-culture CM of scrambled and miR-34a-5p transfected THP-1 (both M0 and M2) and A549 cells; (**G**). Graph showing % proliferation of H1299 cells; (**H**,**I**). % Wound closure of the scratch created in monolayer and photographic images of scratches at different time intervals (0 h, 24 h); (**J**–**L**). Colony counts, photographic images showing colonies, and spectrophotometric determination of clonogenic capacity of H1299 cells upon treatment with same CM. * *p* <0.05, ** *p* < 0.01, *** *p* < 0.001, **** *p* < 0.0001. (**M**). Nitrite content when M0 and M2 macrophages were transfected with scrambled and miR–mimics, respectively; (**N**). Nitrite content upon co-culture of transfected macrophages with A549 cells, as deter. * *p* < 0.05, ** *p* < 0.01, *** *p* < 0.001,**** *p* < 0.0001; (**O**,**P**). Flow cytometric measurement of apoptosis in A549 cells using Annexin V—FITC. CM from co-culture of M2 transfected with scrambled and miR-mimics, respectively, with A549 cells was used to treat A549 and H1299 cells for 48 h. Cells were stained with Annexin–V/FITC and PI, fluorescence of A549 cells was measured using FACS and; (**Q**). Western blotting. CM treated A549 and H1299 cells were lysed, total protein was extracted in RIPA extraction buffer and expression of Procaspase-3 was detected using Western blotting. Protein expression was normalized by expression of β-actin, an endogenous control. * *p* < 0.05, ** *p* < 0.01. Three independent experiments were performed (N = 3). M0-scrambled—unstimulated THP-1 cells transfected with scrambled, M2-scrambled—*IL-4* stimulated THP-1 cells transfected with scrambled, M0-miR-34a-5p—unstimulated THP-1 cells transfected with miR mimic, M2-miR-34a-5p-*IL4* stimulated THP-1 cells transfected with miR mimic.

To elucidate macrophage polarization effects on NSCLC cells, miR-34a-5p mimic transfected macrophages (M0, M2) were co-cultured with A549 cells. The co-culture CM was used to treat A549 and H1299 cells and analyzed for cancer hallmarks. We observed a significant decrease in percent proliferation, wound closure, and number of colonies, upon treatment with CM (from M2 co-culture) in both A549 (Figure 6A–F) and H1299 cells (Figure 6G–L). Additionally, there was a concomitant increase in the concentration of nitrites in CM of both M0 and M2 (miR-transfected) co-culture (Figure 6N). CM treatment from M2 co-culture also increased the percentage of apoptosis in A549 cells (Figure 6O,P). Besides this, an increase in expression of pro-caspase-3 was also observed by Western blotting in both A549 and H1299 cells (Figure 6Q). The relative expression of Caspase-3 was checked by semi-quantitative qRT-PCR (Supplementary Figure S13). These results demonstrate that miR-34a-5p mediated modulation of macrophage polarization reverses the processes of tumorigenesis in NSCLC cells. 

### 3.8. KLF4 and Tumor Inversion in NSCLC Microenvironment 

**Figure 7:**
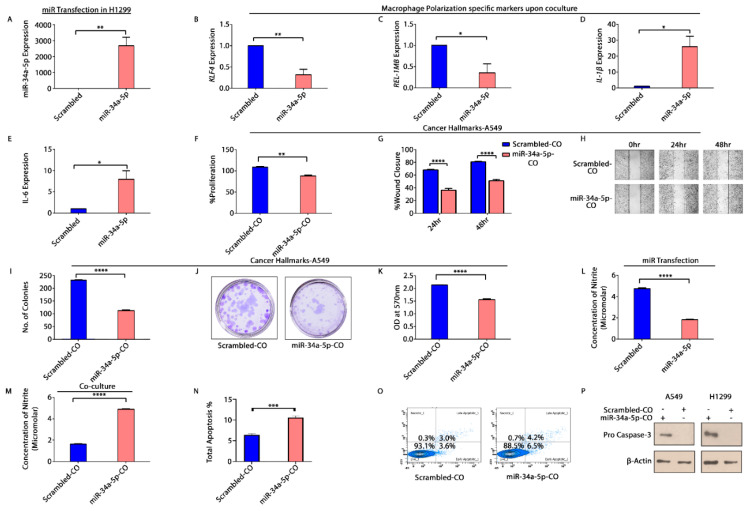
Reversal of TAM polarization and anti-tumor effects in NSCLC microenvironment. miR-34a-5p was overexpressed in H1299 cells. (**A**) Relative fold change in expression of miR-34a-5p upon transfection of Scrambled and miR-mimic in H1299 cells, shown by qRT-PCR; (**B**) relative fold change in expression of *KLF4*; (**C**) *REL-1MB*; (**D**) *IL-1β*; and (**E**) *IL-6* upon co-culture of transfected H1299 cells with THP-1 cells (M2). * *p* < 0.05, ** *p* < 0.01. Cancer hallmarks after co-culture (**F**) cellular proliferation of A549 cells upon treatment with CM of above co-culture (transfected H1299 and M2-THP-1 cells); (**G**,**H**) wound closure of the scratch created in monolayer of A549 cells treated with the same CM; (**I**–**K**) number of colonies counted by ImageJ software, photographic images of colonies and spectrophotometric determination of clonogenic capacity of A549 cells upon treatment with CM. ** *p* < 0.01, *** *p* < 0.001, **** *p* < 0.0001; (**L**) concentration of nitrite released upon transfection of H1299 cells with scrambled and miR-34a-5p mimic; (**M**) concentration of nitrite secreted when transfected H1299 cells were co-cultured with THP-1 cells, as determined by Nitrite assay. *** *p* < 0.001; (**N**,**O**) flow cytometric measurement of apoptosis of A549 cells treated with CM of above co-culture using Annexin V—FITC; (**P**) Western blotting—CM treated cells (A549, H1299) were lysed, total protein was extracted in RIPA extraction buffer and expression of Pro-caspase-3 was detected using Western blotting. Protein expression was normalized by expression of β-Actin, an endogenous control. * *p* < 0.05, ** *p* < 0.01. All the experiments were independently done thrice (N = 3).

To decipher the functional significance of *KLF4* in progression of NSCLC, we overexpressed H1299 cells with control (scrambled) and miR-34a-5p. The effect of transfection on expression of *KLF4* and its regulated genes—*IL-6* and *IL-1β* was checked semi quantitatively (Supplementary Figure S7). The transfected cells were co-cultured with *IL-4* stimulated THP-1 cells. A significant increase in the relative expression of miR-34a-5p was observed upon transfection (Figure 7A). We observed a significant reduction in expression of M2 specific markers—*KLF4* (Figure 7B) and *REL-1MB* (Figure 7C), with a compelling increase in expression of M1 specific markers—*IL-1β* (Figure 7D) and *IL-6* (Figure 7E) upon co-culture. Further, co-culture CM was used to treat A549 cells and analyze the cancer hallmarks. Cellular proliferation, wound healing potential and clonogenic capacity of NSCLC cells was markedly decreased in cells treated with co-culture CM from miR-transfected cells (Figure 7F–K). Moreover, the concentration of nitrites in H1299cells was reduced upon miR-34a-5p transfection (Figure 7L), but was significantly increased upon co-culture (Figure 7M). An increase in apoptosis of A549 cells (Figure 7N,O) and protein levels of pro-caspase3 upon treatment with co-culture CM was also observed (Figure 7P).

## 4. Discussion

The tumor microenvironment has brought great interest in last few years, owing to its impact on initiation, development, progression, and prognosis of cancers. Macrophages are the key components of the immune system that exert tumor-promoting and tumor-inhibiting activities in TME. Clinical data has demonstrated that around 70% of TAMs are M2 type and the remaining ones are M1 which further acquires M2 phenotype during cancer progression [53,54,55]. The exclusive M2-like TAMs are correlated with poor prognosis, evade host immune responses, and promote cell migration and angiogenesis [56,57]. Besides, small (~22 nts long) non-coding RNAs, called miRNAs have also been implicated in almost all cellular processes including cellular, cell cycle, and apoptosis along with immune mechanisms. Thus, it is logical to propose the involvement of miRNAs in modulating components of TME. 

The in silico analysis of NSCLC data available on public databases has displayed the differential expression of more commonly immune system related genes in our study. Pathway enrichment analysis of *IL-1β*, *IL-6*, *TNFα*, and *ARG1* (macrophage polarization related genes) demonstrated their denoting enrichment in cytokine signaling in the immune system (Figure 1A). Further, *KLF4*, *IL-1β*, and miR-34a-5p are associated in the form of an FFL (Figure 1B). Noticeably, *KLF4* is highly upregulated (~3-fold upregulation), while *IL-1β* (2.67-fold downregulation) (Supplementary Table S1) and miR-34a-5p are downregulated (Supplementary Table S2) in NSCLC. Higher expression of *KLF4* and lower expression of miR-34a-5p are correlated with the poor OS of NSCLC patients, indicative of their roles as prognostic biomarkers (Table 2). Later, UALCAN-based analysis in NSCLC advocated that higher *KLF4* and lower *IL-1β* expressions were closely associated with advanced stages of the disease and hence could be potent diagnostic biomarkers. The results presented that *KLF4* expression exhibited a progressive increase in NSCLC from stage 1 to stage 4 and the maximal expression can be found in stage 4 (Figure 2A). Similarly, the expression of *IL-1β* was found to be gradually reduced from stage 1 to stage 4, with the minimum expression in stage 4 (Figure 2C). cBioportal-based somatic mutation analysis of both *KLF4* and *IL-1β* has displayed a combined alteration frequency of 2.8% and 1.8% in LUAD and LUSC, respectively, indicating high mutational burden in *KLF4* (Figure 2G). Furthermore, immune infiltration investigation pinpoint towards *KLF4* mediated infiltration of TAMs in both LUAD and LUSC, particularly, favoring infiltration of M2 macrophages, and negative correlation with NK and B cells suggesting the involvement of *KLF4* in the evasion of immunity. Congruently, *IL-1β*, is critically involved in the infiltration of M1 macrophages (Figure 3). In addition, *KLF4* also displayed negative correlation with tumor purity in both LUAD and LUSC. Tumor purity is defined as the percentage of cancer cells in solid tumors, which may act as a prognostic factor indicator or predictor of chemotherapy benefit. It is an important tool in analyzing the patient’s condition in clinical practices as contamination of normal cells in tumor tissues may subsequently hamper genomic analysis [58,59]. Moreover, cancers with high genomic instability will possess more genomic diversity leading to the formation of more neoantigens and greater infiltration of immune cells [60,61]. This is in concordance with our results where high mutational burden in *KLF4* leads to low tumor purity and hence greater tumor infiltration of immune cells, specifically macrophages. 

Further, we analyzed the association of *KLF4*, *IL-1β*, and miR-34a-5p in macrophage polarization. We found that *KLF4* was upregulated in *IL-4* stimulated M2 subset of macrophages, but *IL-1β* was upregulated in M0 macrophages (Figure 4A,B), however, miR-34a-5p inhibits M2 and promotes M1 subtype of macrophages via directly targeting *KLF4* (Figure 4C–G). Additionally, *KLF4* overexpression increased cell proliferation, migration, and clonogenic capacity, but decreased apoptosis of NSCLC cells, suggesting its oncogenic role within the regulatory network of NSCLC. Moreover, all the above effects were rescued back, when p*KLF4 (FL)* was co-transfected with miR-34a-5p (Figure 5), suggesting the implication of *KLF4* in the tumor-suppressive functions of miR-34a-5p and its oncogenic transformation. The oncogenic nature of *KLF4* in NSCLC is quite contradictory, as most of the studies have depicted the tumor suppressive role of *KLF4* in NSCLC. However, tumor suppressive and oncogenic functions of *KLF4* are cell type, context, and subcellular localization dependent, but how *KLF4* exert these differential functions and the associated molecular mechanisms remains unraveled. Contemporary studies have demonstrated the role of alternative splicing of *KLF4*. Multiple isoforms of *KLF4* have been identified in several cancer types, with *KLF4*α, being most significant in tumorigenesis [62]. *KLF4* transcript is about 3kb long and multiple shorter isoforms (*KLF4α*, *β*, *γ and δ*) have been found. *KLF4 (FL)* (isoform 2) shows a band at around 1440bp; however, *KLF4α* displays a band at 440bp. This isoform lacks exon 3, causing a frameshift in exon 4 to a premature stop codon in exon 5. Moreover, it lacks all the three zinc finger domains of *KLF4 (FL)* and its nuclear localization signal (NLS) sequence, due to which it is mainly located in the cytoplasm, while *KLF4 (FL*) mainly exists in nuclei. Moreover, *KLF4α* interacts with *KLF4 (FL)* and prevents its nuclear translocation, thereby modulating the transcriptional landscape and exerting antagonistic effects on *KLF4 (FL)* [62,63]. Several miRNAs, including miR-34, have been found to regulate *KLF4* post transcriptionally and alter the splicing patterns [64]. Further, it has been found that *KLF4α* is highly expressed in tumors and although *KLF4 (FL)* exerts tumor inhibitory effects, but excessive *KLF4α* opposes its effect, thereby promoting tumorigenesis. This effect of *KLF4α* is seen in Figure 5, when *pc-DNA-KLF4 (FL)* is transfected in A549 cells, it acts as an oncogene. This is because *KLF4α* is highly expressed in A549 cells, which may have masked the *KLF4 (FL)* from nuclear translocation and exert its tumor suppressing effects. However, miR-34a-5p modulates the alternative splicing of *KLF4* and reduces the *KLF4α* isoform, thereby rendering the *KLF4 (FL)* free to exert its tumorigenic effects (Supplementary Figure S7).

Subsequently, to retrospect the significance of macrophage polarization in development and progression of NSCLC, we co-cultured miR-34a-5p transfected macrophages with NSCLC cells and analyzed the effects on cancer hallmarks. There was a concomitant decrease in cellular proliferation, migration and clonogenic capacity of both A549 and H1299, accompanied by an increase in nitrite content and hence apoptosis upon treatment with co-culture CM from macrophages transfected with miR-mimic, suggesting the tumoricidal potential of reprogrammed macrophages (Figure 6). 

Furthermore, to persuade the role of *KLF4* in polarization of TAMs in NSCLC microenvironment, we transfected miR-34a-5p mimic in H1299 cells (Figure 7A). The relative expression of *KLF4* was reduced, and its regulated genes—*IL-1β*, *IL-6* were increased (Supplementary Figure S7). We co-cultured them with *IL-4* stimulated macrophages. We found out a convincing decrease in expression of M2 signature genes-*KLF4*, *REL-1MB* (Figure 7B,C) and *ARG1* (Supplementary Figure S8, nonsignificant), along with an increase in expression of M1 signature genes-*IL-1β*, *IL-6* (Figure 7D,E) and *iNOS* (Supplementary Figure S8). These results thus demonstrate that miR-34a-5p when overexpressed in NSCLC cells, inhibits *KLF4* by degradation of oncogenic *KLF4α* isoform of NSCLC cells, accompanied with an increase in expression of *KLF4* regulated genes—*IL-1β* and *IL-6* (Supplementary Figure S7). Increased secretion of pro-inflammatory mediators *(Il-1β*, *IL-6*) provide a stimulus for polarization of M1 macrophages in co-culture. M1 macrophages, then exert positive feedback to enhance the release of more *IL-1β*, *IL-6* (Figure 7D,E) and *iNOS* (Supplementary Figure S7), upon co-culture. These pro-inflammatory cytokines, however, exert anti-tumor effects, via an autocrine mechanism, as demonstrated by reduced proliferation, wound healing, clonogenic capacity, and enhanced apoptosis of NSCLC cells. 

We also observed a significant decrease in nitrite content of H1299 cells upon transfection of miR-34a-5p, while the content increased upon co-culture of miR-transfected H1299 cells with macrophages (Figure 7L,M). Higher nitrite content upon co-culture leads to increased apoptosis of NSCLC cells. It has been reported that patients with LC exhale high levels of NO and its metabolites—nitrite, and nitrotyrosine, which are associated with advanced stages and poor survival. NO has also been shown to possess both pro and anti-apoptotic activities, based on the cellular context, dosage, and oxidative state of the cells. A high concentration of NO is known to induce apoptosis resistance by S-nitrosylation of FLIP and Bcl2 (*KLF4* regulated anti-apoptotic protein) which makes them resistant to proteasomal degradation and, hence preventing apoptosis. NSCLC cells release high amounts of NO, which can thus be correlated with their aggressive behavior and resistance to apoptosis [53]. However, miR-34a-5p overexpression in NSCLC reduces NO content which would reverse the above effects on Bcl2, thereby presuming apoptosis. However, in case of co-culture with THP-1 cells, we have observed an increase in nitrites along with increased apoptosis. This can be explained as due to miR-34a mediated polarization of M2 TAMs to M1, the expression of *iNOS* also increases. *iNOS* secretes high levels of NO which creates an oxidative state via its activity on mitochondrial membrane permeability. This causes a release of cytochrome c into the cytoplasm, which initiates apoptosis via binding to Apaf and thereby activating caspase 9, caspase 7 and 3, ultimately leading to downstream events of apoptosis [65]. These observations are clear indications of both pro and anti-apoptotic activities of NO based upon dosage and cellular oxidation states. Further, excessive secretion of NO and cytokines like *TNF-α* by M1 macrophages (Supplementary Figures S4 and S5) also activate the immune system, promoting inflammatory responses and generating anti-tumor immunity. 

Overall, our study has suggested two important aspects of TAM polarization in NSCLC. The first aspect is the association of *KLF4* with tumorigenesis in NSCLC. Our results have suggested that *IL-4* mediated induction of *KLF4* not only induces M2 phenotype, but also inhibits the M1 pathway. This was consistent with the observation that *KLF4* deficiency debilitates the ability of *IL-4* to inhibit M1 targets, as demonstrated by an increase in *IL-1β* (M1 gene, when *KLF4* was degraded in *IL-4* stimulated M2 THP-1 cells upon miR-34a-5p transfection (Figure 4F, Supplementary Figure S5)). This is suggestive of the role of *IL-4* mediated cross-talk between TAMs and NSCLC cells, and promotion of tumorigenesis. 

The second aspect is related to *KLF4* mediated regulation of NO content. Tumor cells secrete a large amount of NO, which promotes tumor progression by induction of tumor-cell invasion, proliferation, and the expression of angiogenic factors [55]. However, immune cells present in TME, like macrophages, when polarized to M1 state upregulates *iNOS* (Supplementary Figure S5), leading to an accumulation of more NO. This macrophage secreted NO exerts tumoricidal activity due to which host *iNOS* might suppress tumor growth and metastasis. Our study has also demonstrated the potential of miR-34a-5p to polarize TAMs from M2 to M1 phenotype by directly targeting *KLF4* in the LC microenvironment. Consequently, these reprogrammed M1 macrophages exhibit cytotoxic effects on NSCLC cells via NO associated processes (Figure 7). Although NO, mediated apoptosis is the mechanism by which M1 macrophages have exerted their tumoricidal actions here, but there are several other factors such as MMP induction and decreased secretion of growth factors, that are also accountable and need to be further explored. Additionally, a better understanding of the circuits associated with inflammatory cytokines, other growth factors, and miRNAs will aid in unravelling the molecular mechanisms associated with macrophage polarization in TME, which would pave a way towards the development of novel and more efficient therapeutic strategies for NSCLC. 

## Figures and Tables

**Table 1:** Summary of regulatory relationships among NSCLC-specific miRNAs, DEGs, and TFs.

Relationship	No. of Edges	No. of miRNAs	No. of TFs	No. of Genes
miRNA-gene ^a^	22	22	-	4
TF-gene ^b^	37	-	23	4
miRNA-TF ^c^	183	22	23	-

^a^ miRNA-gene: miRNA repression of genes, ^b^ TF-gene: TF regulation of genes, ^c^ miRNA-TF: miRNA repression of TFs.

**Table 2:** Median OS time in lower and higher expression cohorts in TCGA-LUAD cohort.

Items	Low Expression Cohort (Months)	High Expression Cohort (Months)
*KLF4*	51.03	34.77
*IL-1β* (ns)	42.27	59.27
miR-34a-5p	48.47	59.27

## Data Availability

The datasets used in our study were downloaded from Gene Expression Omnibus (GEO) under accession numbers GSE75037 (https://www.ncbi.nlm.nih.gov/geo/query/acc.cgi?acc=GSE75037, accessed on 15 March 2021) and GSE53882 (https://www.ncbi.nlm.nih.gov/geo/query/acc.cgi?acc=GSE53882, accessed on 15 March 2021).

## Supplementary Materials

The following are available online at https://www.mdpi.com/article/10.3390/cells10082091/s1, Supplementary Figures, Figure S1: NSCLC-specific 3-node miRNA–FFL regulatory network comprising 49 nodes and 242 edges, Figure S2: Topological Graphs, Figure S3: Scatter plots exhibiting correlations of *KLF4* with infiltrating levels of immune cells, Figure S4: Macrophage stimulation, Figure S5: Relative expression of M1 macrophage specific markers, Figure S6: Relative expression of M2 macrophage specific markers, Figure S7: Relative expression of *KLF4*, *IL-1β* and *IL-6* upon transfection of NSCLC cells with Scrambled and miR-34a-5p, Figure S8: Differential expression of macrophage specific markers, upon Co-culture of M2 macrophages with transfected H1299 cells, Figure S9: Western Blotting, Figure S10: SSC/FSC plots of Flow cytometry based apoptotic analysis using AnnexinV /FITC—PI staining of A549 cells transfected with pcDNA 3.1, pcDNA 3.1 + *KLF4* and pcDNA 3.1+ *KLF4* + miR, Figure S11: SSC/FSC plots of Flow cytometry based apoptotic analysis using AnnexinV /FITC—PI staining of A549 cells treated with Co-culture Conditioned medium of M2 macrophages (THP-1) cells transfected with Scrambled and miR-34a-5p mimic, Figure S12: SSC/FSC plots of Flow cytometry based apoptotic analysis using AnnexinV /FITC—PI staining of A549 cells treated with Co-culture Conditioned medium of H1299 cells transfected with Scrambled and miR-34a-5p mimic, Figure S13: Relative expression of Caspase-3; Supplementary Tables, Table S1: differentially expressed genes (DEGs) in NSCLC, Table S2: Differentially expressed miRNAs (DEMs) in NSCLC; Oligonucleotide sequences of primers and list of antibodies used.
