# Supplementary material for: Comprehensive Integrative Analysis Reveals the Association of KLF4 with Macrophage Infiltration and Polarization in Lung Cancer Microenvironment
Source: Cells. 2021 Aug 14;10(8):2091. doi: 10.3390/cells10082091 (PMC8392240; doi:10.3390/cells10082091)
Supplement: Supplementary file 1 [file cells-10-02091-s001.zip › cells-1312603-supplementary/SUPPLEMENTARY/OLIGONUCLEOTIDE SEQUENCES.pdf]

The sequences of oligonucleotides used are:

miR-34a-5p mimic - UGGCAGUGUCUUAGCUGGUUGU (Qiagen)

scrambled miRNA mimic - sequence not available (Qiagen, Lot no.#300637469)

*GAPDH*, forward, 5′-GGATTTGGTCGTATTGGG-3′ and reverse, 5′-GGAAGATGGTGATGGGATT-3; [1]

*KLF4,* forward, 5'-GTGCCCCGACTAACCGTTG-3', and reverse primer 5'-GTCGTTGAACTCCTCGGTCT-3′′; [2] *(KLF4α)*

*KLF4,* forward, 5'-ACGATCGTGGCCCCGGAAAAGGAC-3', and reverse primer 5'-CAACAACCGAAAATGCACCAGCCCCAG-3′′; [3] *(KLF4-Total)*

*REL-1MB,* forward, 5′-GCAAGAAGCTCTCGTGTG CTAG-3′,and reverse, 5′-AACATCCCACGAACCACAGCCA-3′; [4]

*IL-6*, forward, 5′- TCATCACTGGTCTTTTGGAG-3′ and reverse, 5′-GTCAGGGGTGGTTATTGC-3′; [5]

*IL-1β,* forward, 5′-ACAGATGAAGTGCTCCTTCCA-3′ and reverse, 5′-GTCGGAGATTCGTAGCTGGAT-3′; [6]

*INOS,* forward, 5′- CAGCGGGATGACTTTCCAAG-3′ and reverse, 5′-AGGCAAGATTTGGACCTGCA-3′; [7]

*TNF-α,* forward, 5'- ATGGGCTACAGGCTTGTCACTC-3'and reverse, 5'-CTCTTCTGCCTGCTGCACTTTGTNF-3'; [8]

*ARG1*, forward, 5'-GGCAAGGTGATGGAAGAAAC-3', and reverse 5'-AGTCCGAAACAAGCCAAGGT-3'; [7]

*Caspase-3*, forward, 5'-ATGGGAGCAAGTCAGTGGAC-3', and reverse 5' CGTACCAGAGCGAGATGACA-3'; [9]

**LIST OF ANTIBODIES USED:**

**PRIMARY ANTIBODY -** Rabbit Caspase 3 (H-277), Santa Cruz Biotechnology, USA(Cat # sc-7148); Rabbit KLF4, Invitrogen, USA (Cat # PA5-20897); Rabbit Arg, Cloud clone, USA (Cat # PAB120Mu01); Mouse β-actin (C4), Santa Cruz Biotechnology, USA (Cat # sc-47778)

**SECONDARY ANTIBODY -** HRP conjugated Mouse anti-rabbit IgG (Cat # sc-2357) and Rabbit anti-mouse IgG-κ, Santa Cruz Biotechnology, USA (Cat # sc-358914)

## REFERENCES


1. Attia, Y.M., et al., *The FXR agonist, obeticholic acid, suppresses HCC proliferation & metastasis: role of IL-6/STAT3 signalling pathway.* Scientific reports, 2017. 7(1): p. 1-12.
2. Tang, G., et al., *Transcriptional repression of FOXO1 by KLF4 contributes to glioma progression.* Oncotarget, 2016. 7(49): p. 81757.
3. Kim, Y.M., et al., *Effects of mechanical stimulation on the reprogramming of somatic cells into human-induced pluripotent stem cells.* Stem cell research & therapy, 2017. 8(1): p. 1-12.
4. Jin, H., et al., *Investigating resistin like beta (RETNLB) as a tumor promoter for oral squamous cell carcinoma.* Head & Face Medicine, 2021. 17(1): p. 1-10.
5. Zhang, S., et al., *Tumor-associated macrophages promote tumor metastasis via the TGF-β/SOX9 axis in non-small cell lung cancer.* Oncotarget, 2017. 8(59): p. 99801.
6. Lei, H., et al., *Human S100A7 induces mature interleukin1α expression by RAGE-p38 MAPK-calpain1 pathway in psoriasis.* PLoS One, 2017. 12(1): p. e0169788.
7. Jung, K., et al., *Elevated ARG1 expression in primary monocytes-derived macrophages as a predictor of radiation-induced acute skin toxicities in early breast cancer patients.* Cancer biology & therapy, 2015. 16(9): p. 1281-1288.
8. Devarapu, S.K., et al., *Tumor necrosis factor superfamily ligand mRNA expression profiles differ between humans and mice during homeostasis and between various murine kidney injuries.* Journal of biomedical science, 2017. 24(1): p. 1-11.
9. Yang, C., et al., *Transcriptional activation of caspase-6 and-7 genes by cisplatin-induced p53 and its functional significance in cisplatin nephrotoxicity.* Cell Death & Differentiation, 2008. 15(3): p. 530-544.
