# Supplementary material for: Comprehensive Integrative Analysis Reveals the Association of KLF4 with Macrophage Infiltration and Polarization in Lung Cancer Microenvironment
Source: Cells. 2021 Aug 14;10(8):2091. doi: 10.3390/cells10082091 (PMC8392240; doi:10.3390/cells10082091)
Supplement: Supplementary file 1 [file cells-10-02091-s001.zip › cells-1312603-supplementary/SUPPLEMENTARY/SUPPLEMENTARY FIGURES.pdf]

SUPPLEMENTARY FIGURES

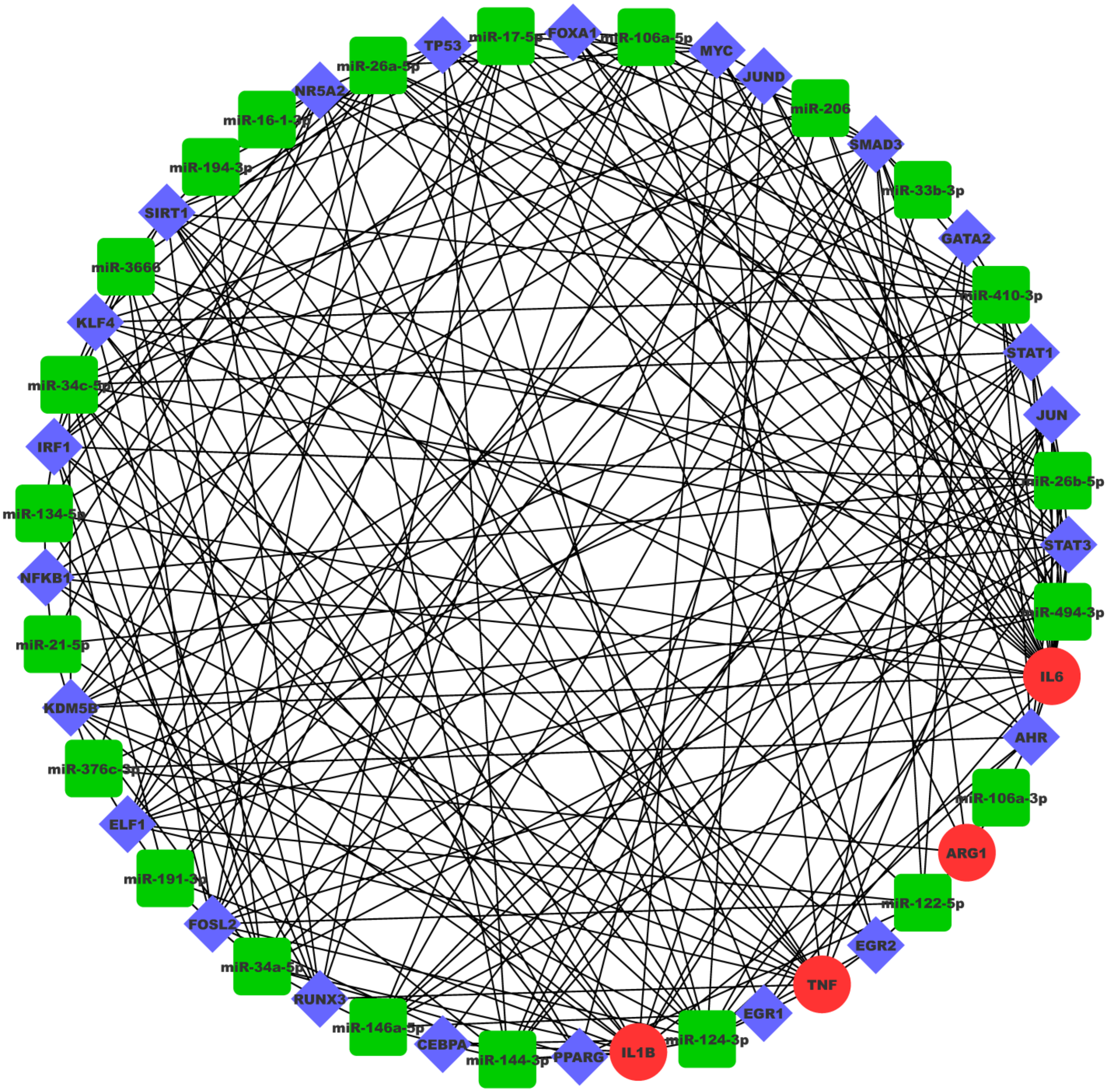

Fig.S1 NSCLC-specific 3-node miRNA–FFL regulatory network comprising 49 nodes and 242 edges. *The green rectangular nodes represent NSCLC-specific miRNAs, red circular nodes represent NSCLC-specific DEGs, and blue diamond nodes represent NSCLC-specific TFs.*

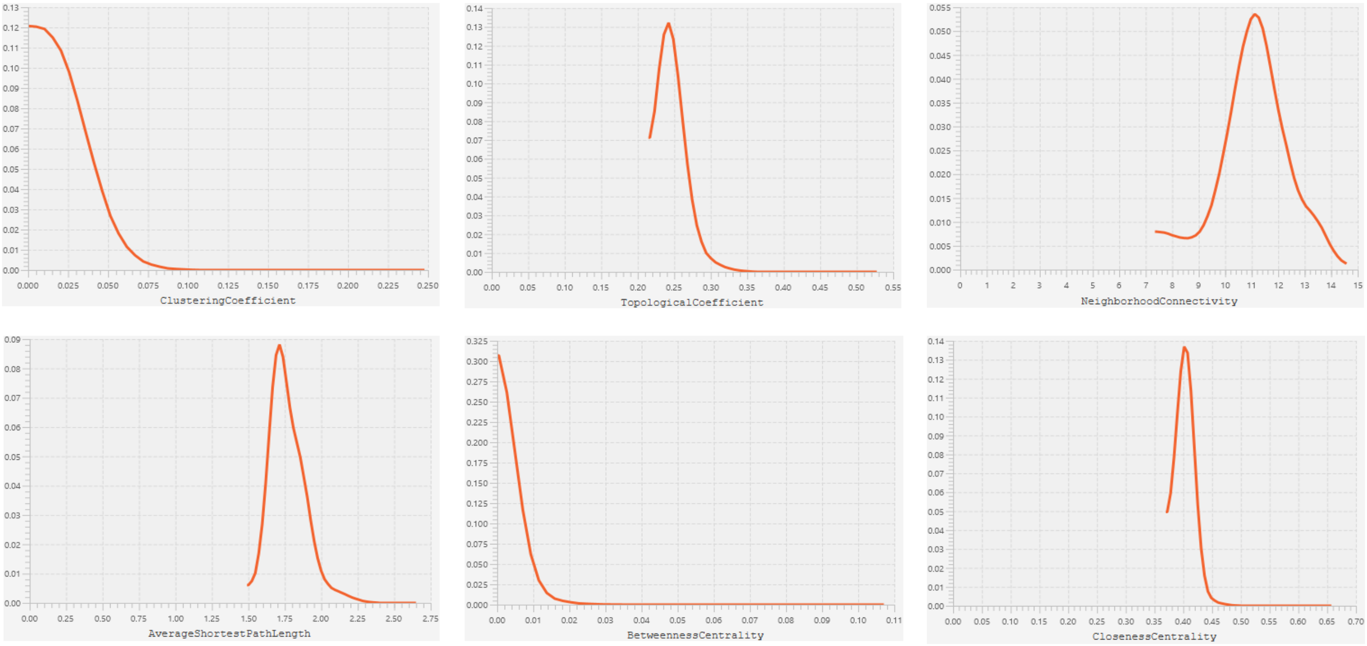

Fig.S2 Topological Graphs. *Topological property / centrality plots of the NSCLC-specific 3-node miRNA FFL representing (A) Clustering coefficient, (B) Topological coefficient, (C) Neighborhood connectivity, (D) Average shortest path length, (E) Betweenness centrality, and (F) Closeness centrality as a function of degree. The lines are fitted with power laws.*

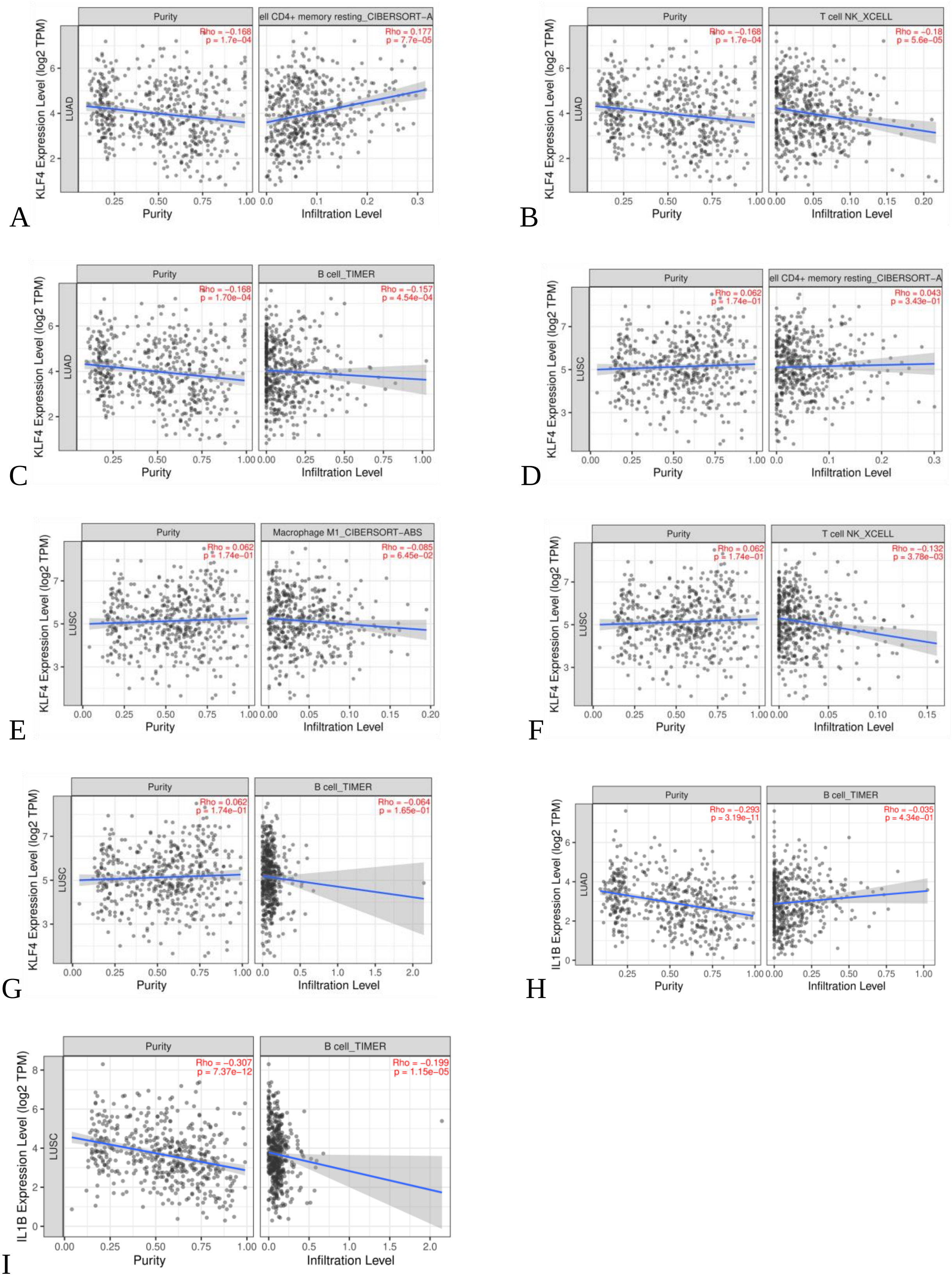

Fig.S3 Scatter plots exhibiting correlations of KLF4 with infiltrating levels of immune cells. *(A) T Cell CD4+, (B) NK cell, and (C) B cell in case of LUAD and (D) T Cell CD4+, (E) M1 Macrophage, (F) NK cell and (G) B Cell in LUSC. Scatter plots showing correlations of IL-1β with infiltrating levels of B cell in case of (H) LUAD and (I) LUSC, respectively. Left and right panel demonstrate gene expression levels against tumor purity and infiltrating levels of immune cells, respectively. In addition, Spearman's correlation value and estimated statistical significance were shown as the legends for each scatter plot.*

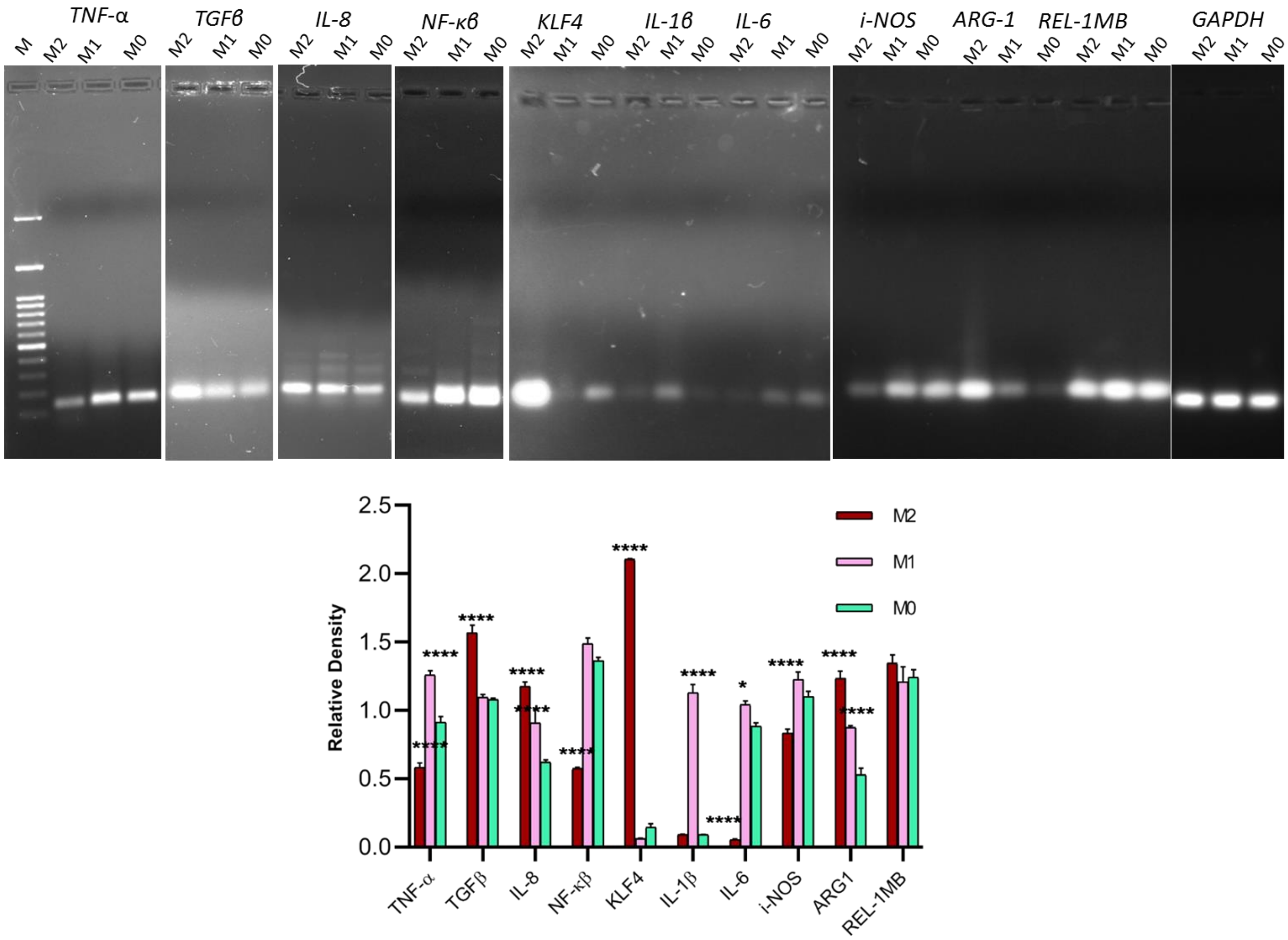

Fig.S4 Macrophage stimulation. *The relative expression of M1 specific markers – TNF-α, NF-κβ, IL-1β, IL-6, i-NOS; and M2 specific markers – TGFβ, IL-8, KLF4, ARG1 and REL-1MB were checked upon stimulation of macrophages via LPS (100ng/ml, L4524, Lipopolysaccharide from E.coli 055:B5, sigma, Saint Louis, MO, USA) and IL-4 (10ng/ml) in M1 and M2 states via semi-quantitative RT-PCR. GAPDH was used as an endogenous control. The unstimulated state M0 was used as a control to compare the expression with M1 and M2 states.* *p < 0.05, **p < 0.01, ***p < 0.001,****p<0.0001.

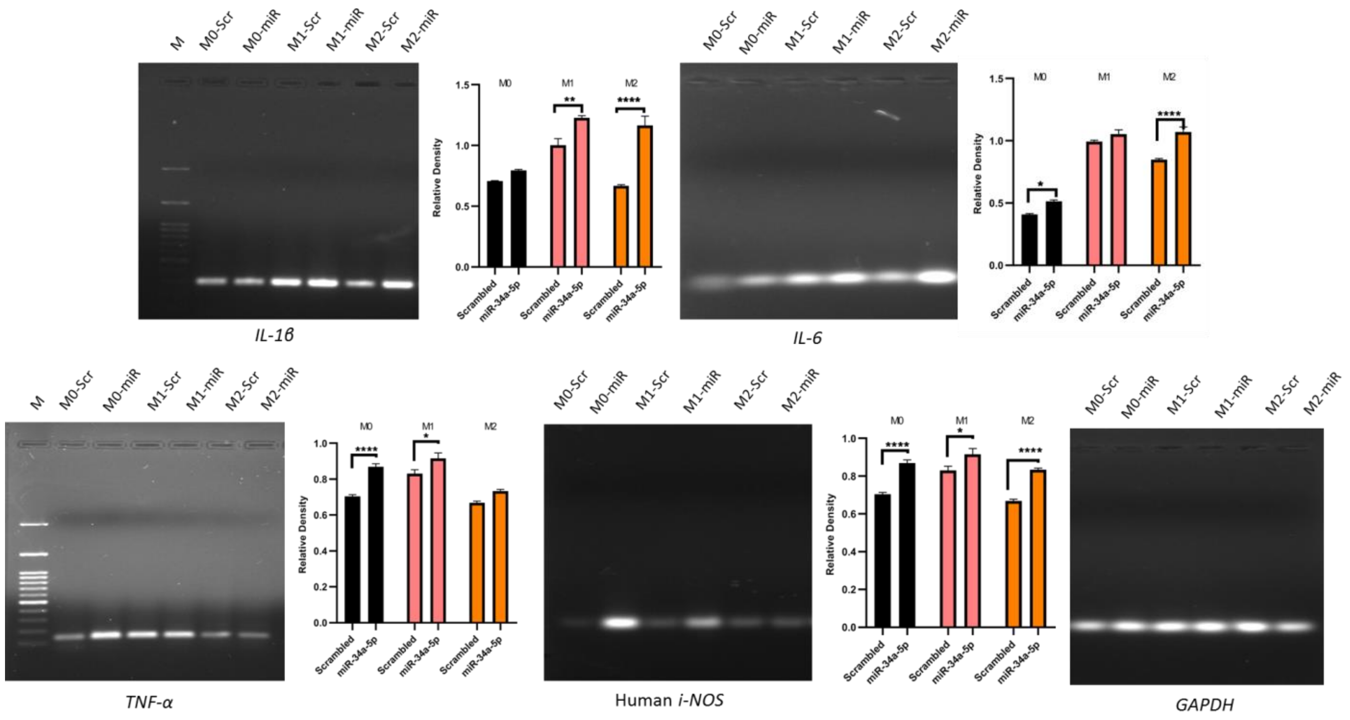

Fig.S5 Relative expression of M1 macrophage specific markers *(IL-1β, IL-6, TNF-α and Human iNOS), upon transfection of THP-1 macrophages (3 subsets- M0, M1 and M2) with Scrambled and miR-34a-5p. The expression was determined via semi-quantitative RT-PCR and relative densities of the bands are plotted. GAPDH was used as an endogenous control. (Scr-scrambled transfection, miR-mir-34a-5p transfection).* *$p < 0.05$, **$p < 0.01$, ***$p < 0.001$,****$p<0.0001$.

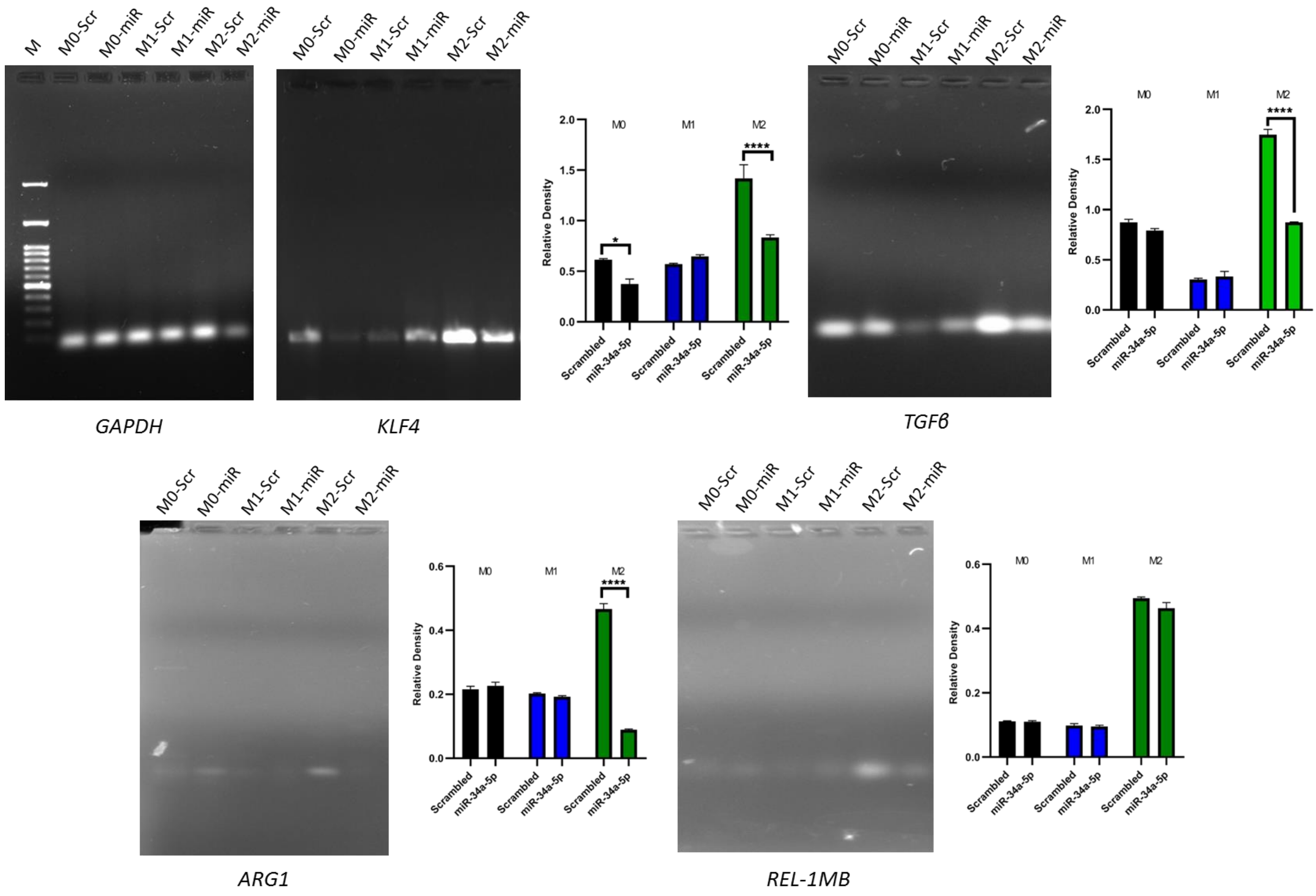

Fig.S6 Relative expression of M2 macrophage specific markers *(KLF4, TGFβ, ARG1 and REL-1MB), upon transfection of THP-1 macrophages (M0, M1 and M2) with Scrambled and miR-34a-5p. The expression was determined via semi-quantitative RT-PCR and relative densities of the bands are plotted. GAPDH was used as an endogenous control. (Scr-scrambled transfection, miR-mir-34a-5p transfection).* *p < 0.05, **p < 0.01, ***p < 0.001,****p<0.0001.

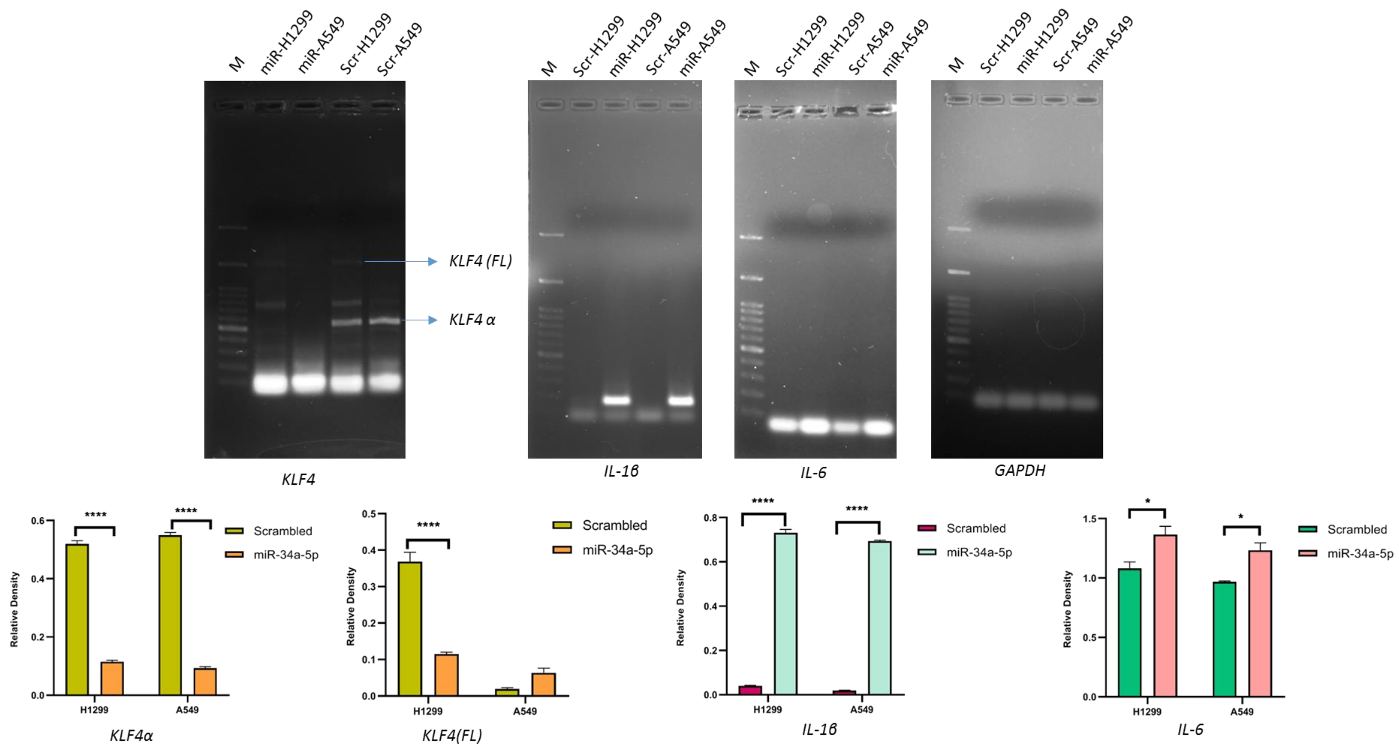

Fig.S7 Relative expression of KLF4, IL-1β and IL-6 upon transfection of NSCLC cells with Scrambled and miR-34a-5p. *The expression was determined via semi-quantitative RT-PCR and relative densities of the bands are plotted. GAPDH was used as an endogenous control. (Scr-scrambled transfection, miR-mir-34a-5p transfection).* *p < 0.05, **p < 0.01, ***p < 0.001,****p<0.0001.

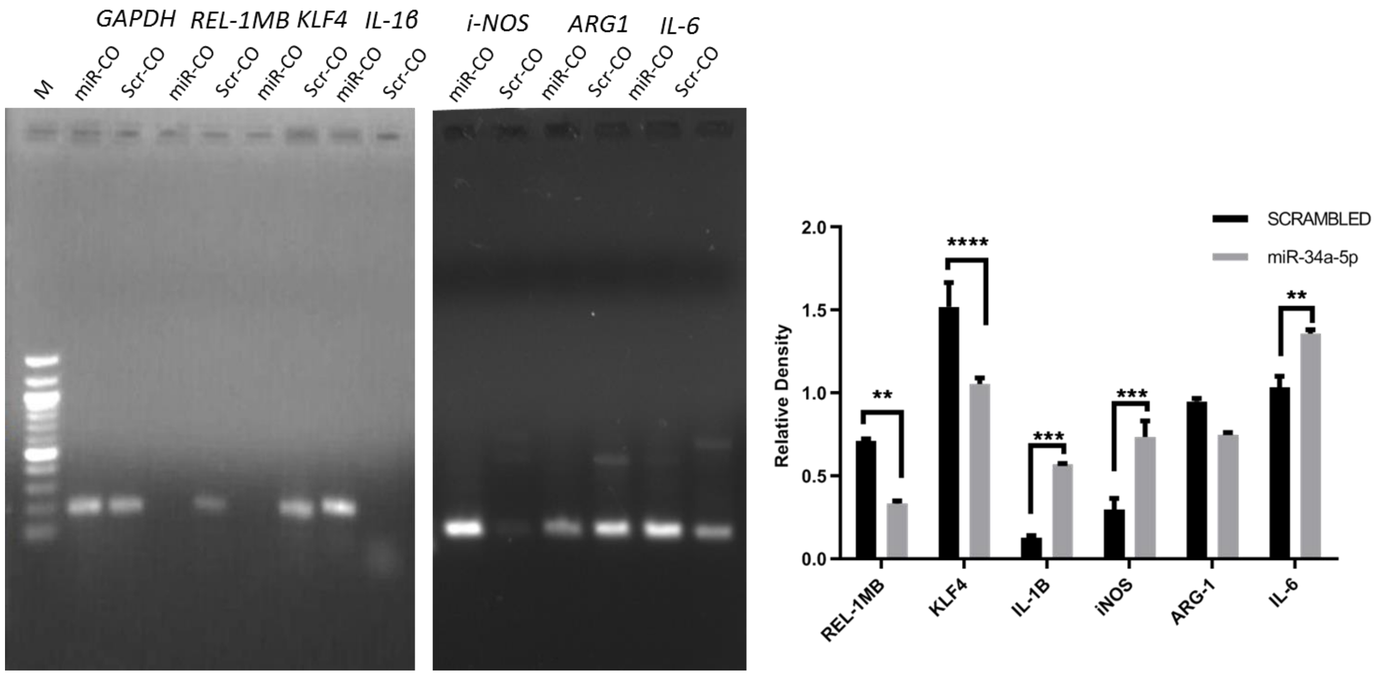

Fig.S8 Differential expression of macrophage specific markers, upon Co-culture of M2 macrophages with transfected H1299 cells. *The expression was determined via semi-quantitative RT-PCR and relative densities of the bands are plotted. GAPDH was used as an endogenous control. (miR-CO -miR transfected cells co-culture, Scr-CO- scrambled transfected cells co-culture).* *p < 0.05, **p < 0.01, ***p < 0.001,****p<0.0001.

(A)

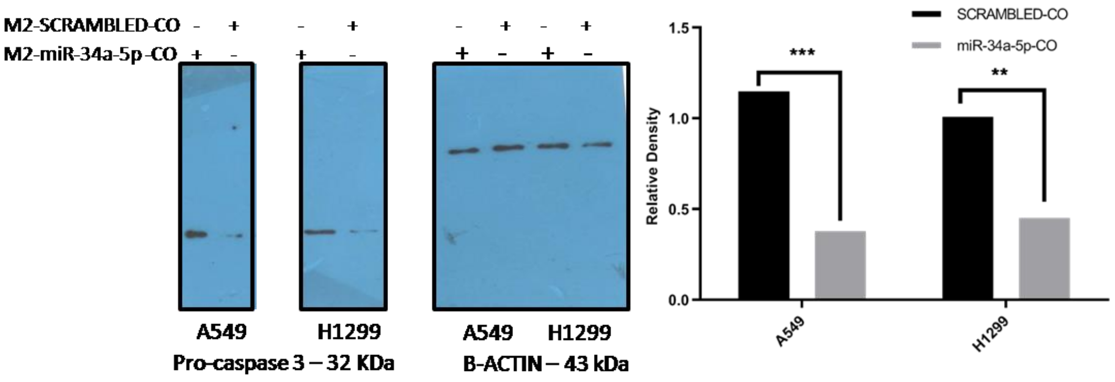

(B)

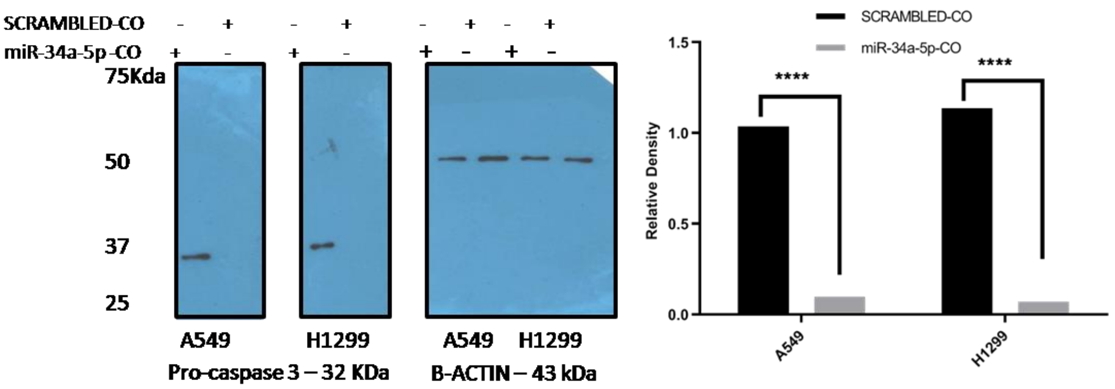

(C)

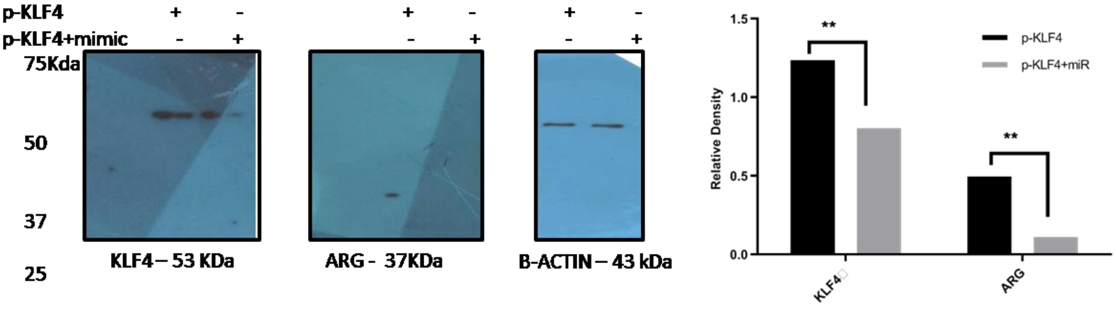

Fig.S9 Western Blotting *(A) Blots and relative densitometry for* Procaspase-3 *and* β-Actin *(endogenous control) after treatment of A549 and H1299 cells with conditioned medium from co-culture of scrambled and miR-34a-5p transfected THP-1(IL-4 stimulated) and A549 cells; (B) Blots and relative densitometry for* Procaspase-3 *and* β-Actin *after treatment of A549 and H1299 cells with conditioned medium from co-culture of scrambled and miR-34a-5p transfected H1299 and THP-1 cells (IL-4*

stimulated); (C) Blots and relative densitometry for KLF4, ARG1 *and* β-actin *developed after overexpression of KLF4 and KLF4+ miR-34a-5p mimic in THP-1 cells.* *p < 0.05, **p < 0.01, ***p < 0.001,****p<0.0001

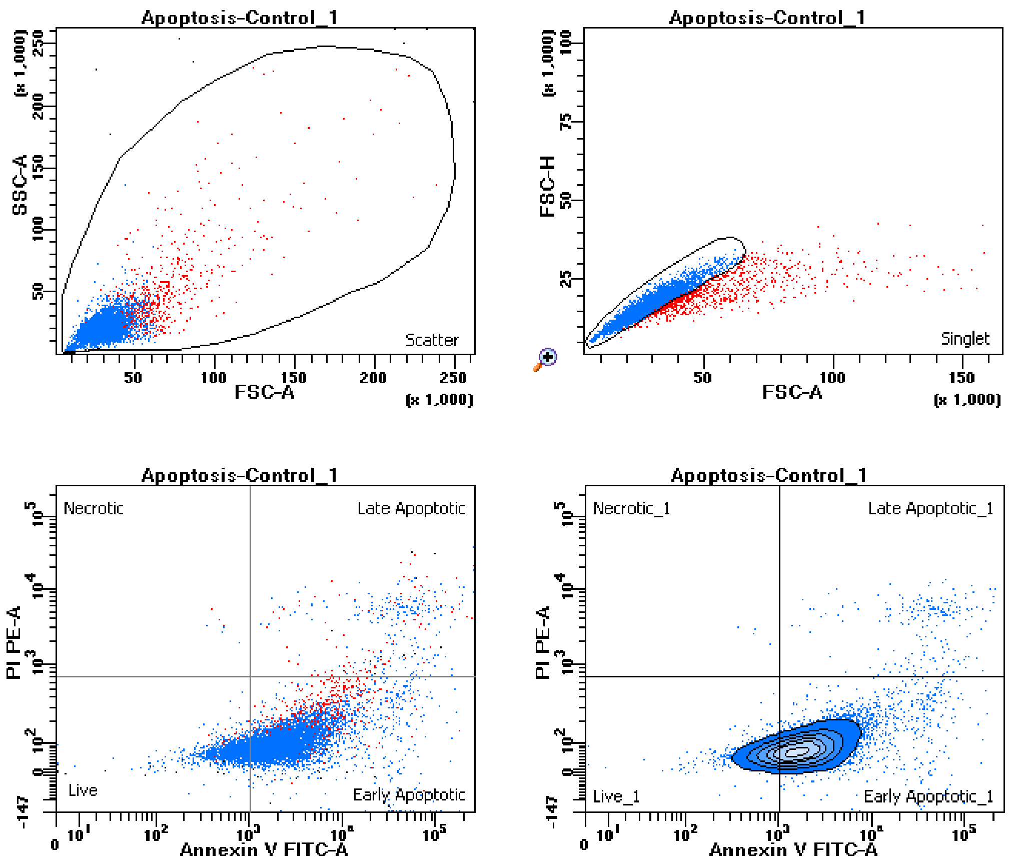

Tube: Control_1

Population	#Events	%Parent	%Total
All Events	10,000	####	100.0
Scatter	9,950	99.5	99.5
Singlet	8,697	87.4	87.0
Necrotic	4	0.0	0.0
Late Apoptotic	214	2.5	2.1
Live	1,663	19.1	16.6
Early Apoptotic	6,816	78.4	68.2
Necrotic_1	4	0.0	0.0
Late Apoptotic_1	213	2.4	2.1
Live_1	1,667	19.2	16.7
Early Apoptotic_1	6,813	78.3	68.1

Experiment Name: Apoptosis_03Feb2020
Tube Name: Control_1

Population	#Events	%Parent	Annexin V FIT... Median	PI PE-A Median
All Events	10,000	####	1,979	89
Scatter	9,950	99.5	1,979	89
Singlet	8,697	87.4	1,856	84
Necrotic	4	0.0	513	2,793
Late Apoptotic	214	2.5	41,156	4,734
Live	1,663	19.1	717	64
Early Apoptotic	6,816	78.4	2,170	90
Necrotic_1	4	0.0	513	2,793
Late Apoptotic_1	213	2.4	41,158	4,770
Live_1	1,667	19.2	718	64
Early Apoptotic_1	6,813	78.3	2,170	90

Fig.S10. (A) SSC/FSC plots *of Flow cytometry based apoptotic analysis using AnnexinV /FITC – PI staining of A549 cells transfected with pcDNA 3.1 for 48 h (Fig.5H)*

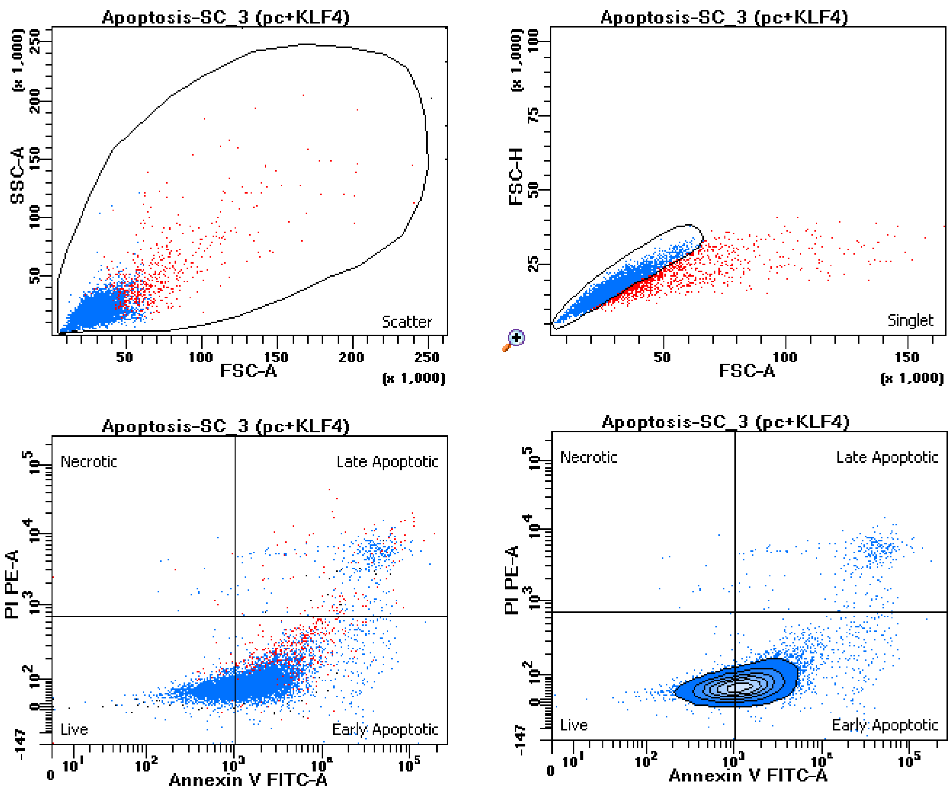

Tube: SC_3 (pc+KLF4)

Population	#Events	%Parent	%Total
All Events	10,000	####	100.0
Scatter	9,947	99.5	99.5
Singlet	8,718	87.6	87.2
Necrotic	14	0.2	0.1
Late Apoptotic	283	3.2	2.8
Live	3,067	35.2	30.7
Early Apoptotic	5,354	61.4	53.5

Experiment Name: Apoptosis_03Feb2020
Tube Name: SC_3 (pc+KLF4)
Record Date: Feb 3, 2020 4:20:02 PM

Population	#Events	%Parent	Annexin V FIT... Median	PI PE-A Median	Time Median
All Events	10,000	####	1,432	70	542
Scatter	9,947	99.5	1,431	70	542
Singlet	8,718	87.6	1,331	66	542
Necrotic	14	0.2	387	1,532	323
Late Apoptotic	283	3.2	35,535	4,865	526
Live	3,067	35.2	663	53	522
Early Apoptotic	5,354	61.4	1,844	74	559

***Fig.S10 (B) SSC/FSC plots** of Flow cytometry based apoptotic analysis using AnnexinV /FITC – PI staining of A549 cells transfected with pcDNA 3.1+ KLF4 for 48 h (Fig.5H).*

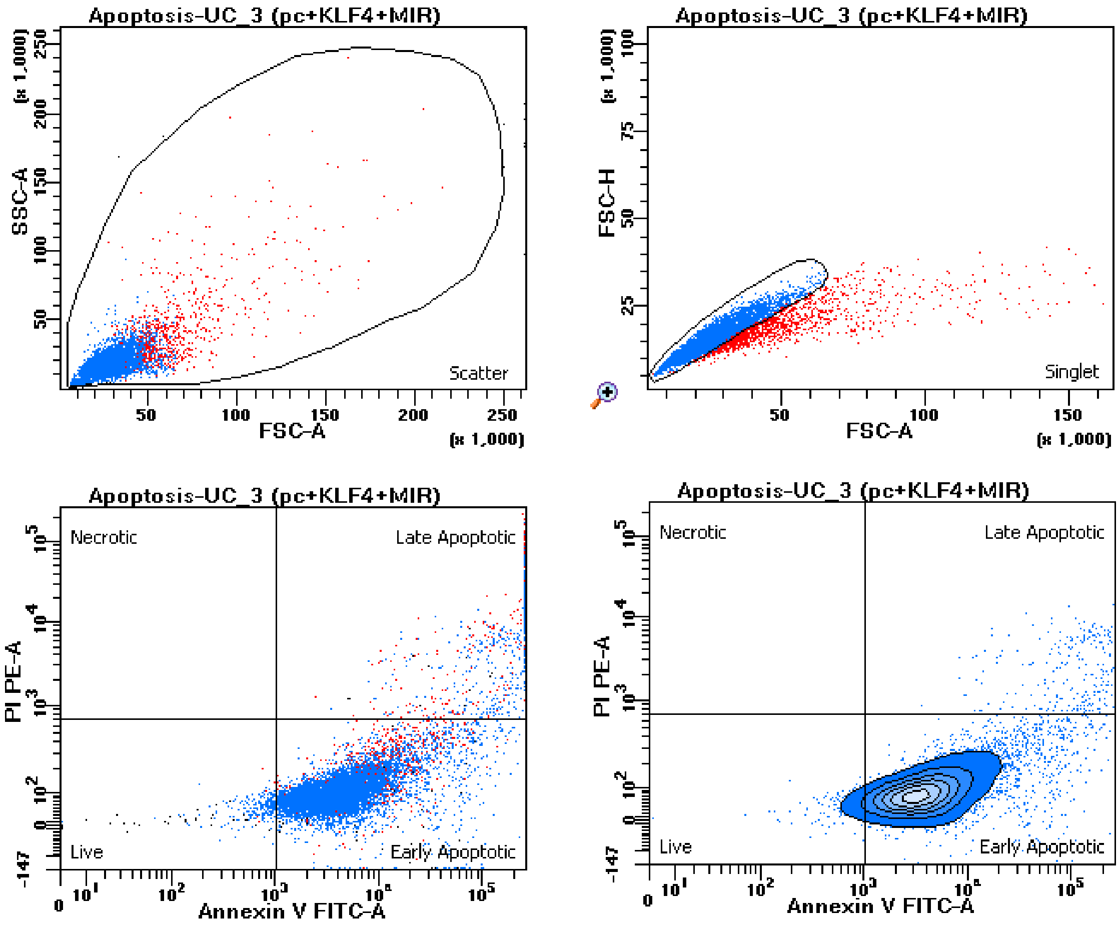

Tube: UC_3 (pc+KLF4+MIR)

Population	#Events	%Parent	%Total
All Events	10,000	#####	100.0
Scatter	9,897	99.0	99.0
Singlet	8,385	84.7	83.9
Necrotic	0	0.0	0.0
Late Apoptotic	368	4.4	3.7
Live	241	2.9	2.4
Early Apoptotic	7,776	92.7	77.8

Experiment Name: Apoptosis_03Feb2020
Tube Name: UC_3 (pc+KLF4+MIR)
Record Date: Feb 3, 2020 4:40:30 PM

Population	#Events	%Parent	Annexin V FIT... Median	PI PE-A Median	Time Median
All Events	10,000	#####	4,353	96	869
Scatter	9,897	99.0	4,352	96	866
Singlet	8,385	84.7	3,988	90	878
Necrotic	0	0.0	#####	#####	#####
Late Apoptotic	368	4.4	188,036	4,837	806
Live	241	2.9	777	51	744
Early Apoptotic	7,776	92.7	3,944	89	883

***Fig.S10 (C) SSC/FSC plots** of Flow cytometry based apoptotic analysis using AnnexinV /FITC – PI staining of A549 cells transfected with pcDNA 3.1+ KLF4+miR for 48 h (Fig.5H).*

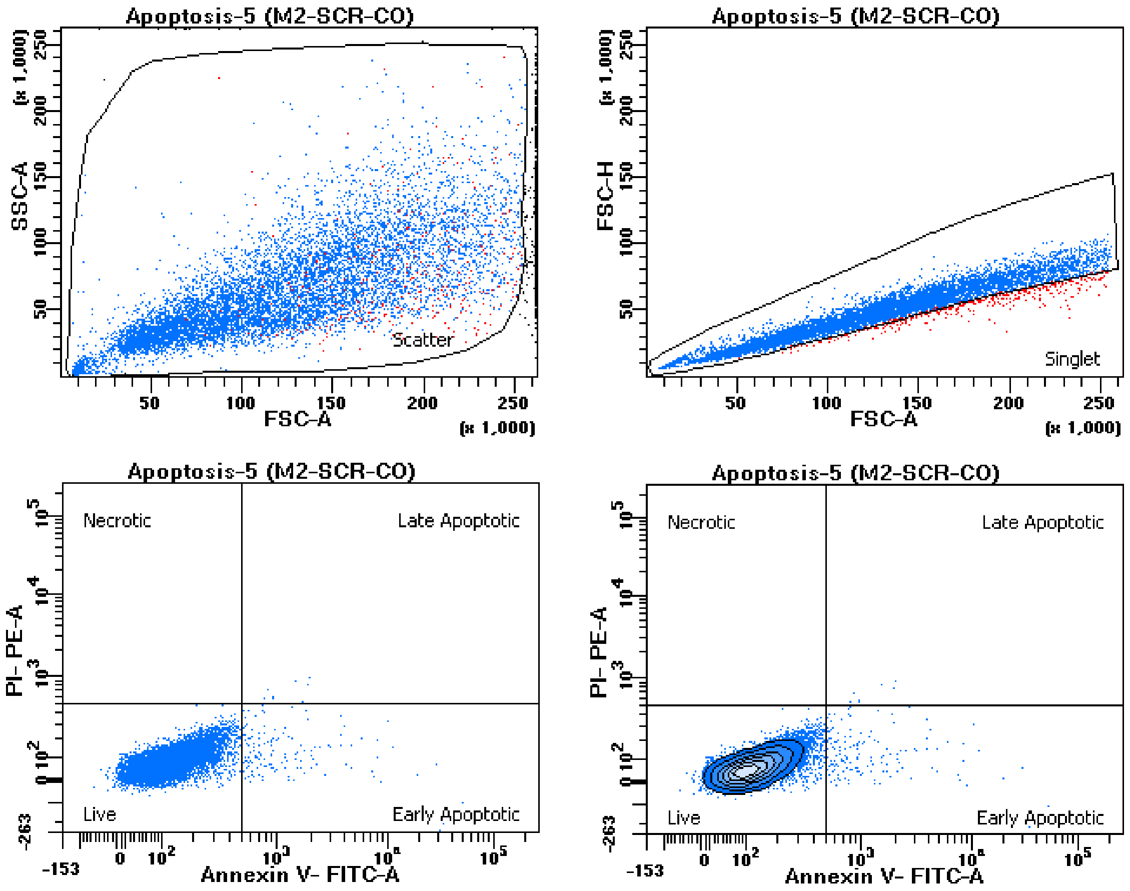

Tube: 5 (M2-SCR-CO)

Population	#Events	%Parent	%Total
All Events	10,000	#####	100.0
Scatter	9,471	94.7	94.7
Singlet	9,015	95.2	90.1
Necrotic	0	0.0	0.0
Late Apoptotic	8	0.1	0.1
Live	8,862	98.3	88.6
Early Apoptotic	145	1.6	1.5

Experiment Name: 19Sept2019_Apoptosis
Tube Name: 5 (M2-SCR-CO)
Record Date: Sep 19, 2019 4:08:05 PM

Population	#Events	%Parent	Annexin V- ... Median	PI- PE-A Median	Time Median
Scatter	9,471	94.7	118	76	704
Singlet	9,015	95.2	116	74	704
Necrotic	0	0.0	#####	#####	#####
Late Apoptotic	8	0.1	1,182	652	1,073
Live	8,862	98.3	115	74	704
Early Apoptotic	145	1.6	868	115	709

***Fig.S11 (A) SSC/FSC plots** of Flow cytometry based apoptotic analysis using AnnexinV /FITC – PI staining of A549 cells treated with Co-culture Conditioned medium of M2 macrophages (THP-1) cells transfected with Scrambled for 48 h (Fig.6P).*

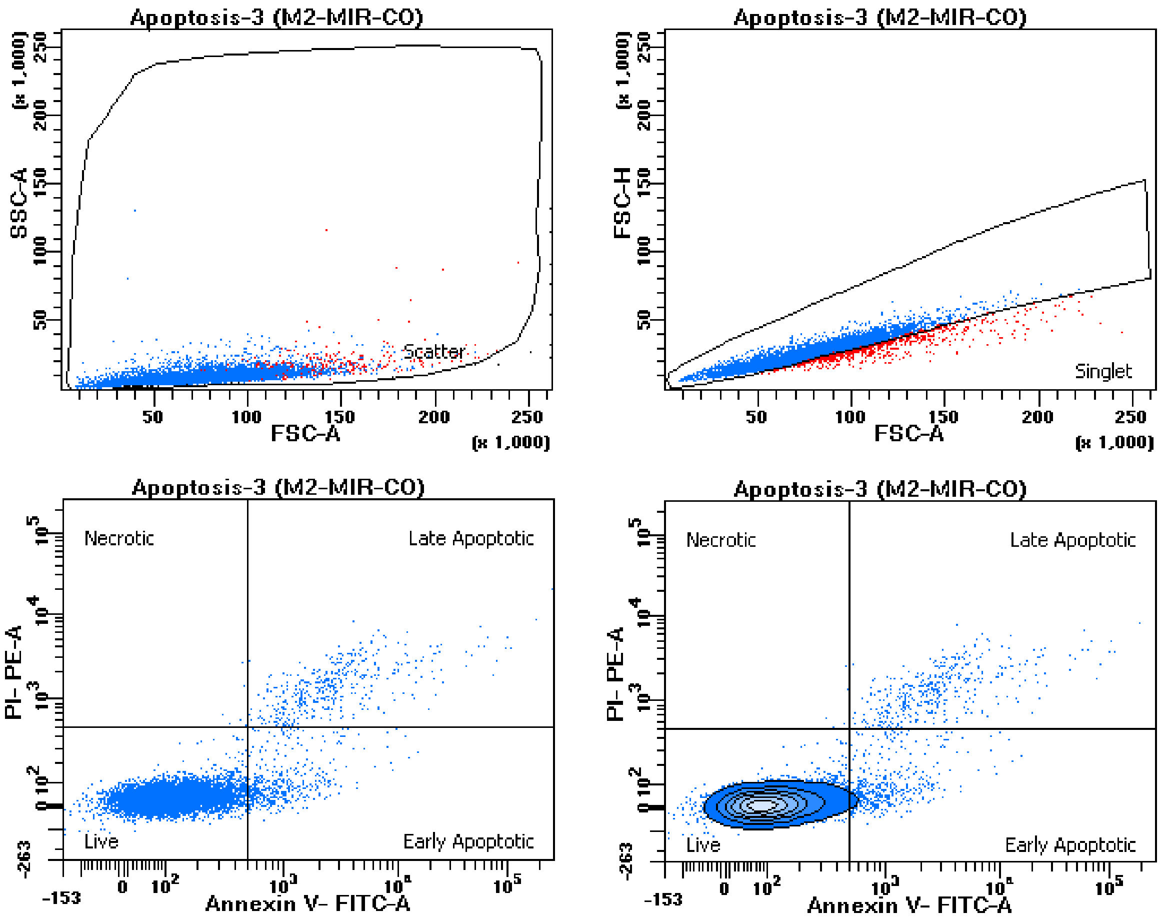

Tube: 3 (M2-MIR-CO)

Population	#Events	%Parent	%Total
All Events	10,000	#####	100.0
Scatter	9,936	99.4	99.4
Singlet	8,732	87.9	87.3
Necrotic	6	0.1	0.1
Late Apoptotic	413	4.7	4.1
Live	7,867	90.1	78.7
Early Apoptotic	446	5.1	4.5

Experiment Name: 19Sept2019_Apoptosis
Tube Name: 3 (M2-MIR-CO)
Record Date: Sep 19, 2019 3:25:11 PM

Population	#Events	%Parent	Annexin V- ... Median	PI- PE-A Median	Time Median
Scatter	9,936	99.4	116	35	472
Singlet	8,732	87.9	112	33	479
Necrotic	6	0.1	434	634	384
Late Apoptotic	413	4.7	2,476	1,381	479
Live	7,867	90.1	100	29	482
Early Apoptotic	446	5.1	821	75	426

***Fig.S11 (B) SSC/FSC plots** of Flow cytometry based apoptotic analysis using AnnexinV /FITC – PI staining of A549 cells treated with Co-culture Conditioned medium of M2 macrophages (THP-1) cells transfected with miR-34a-5p mimic for 48 h (Fig.6P).*

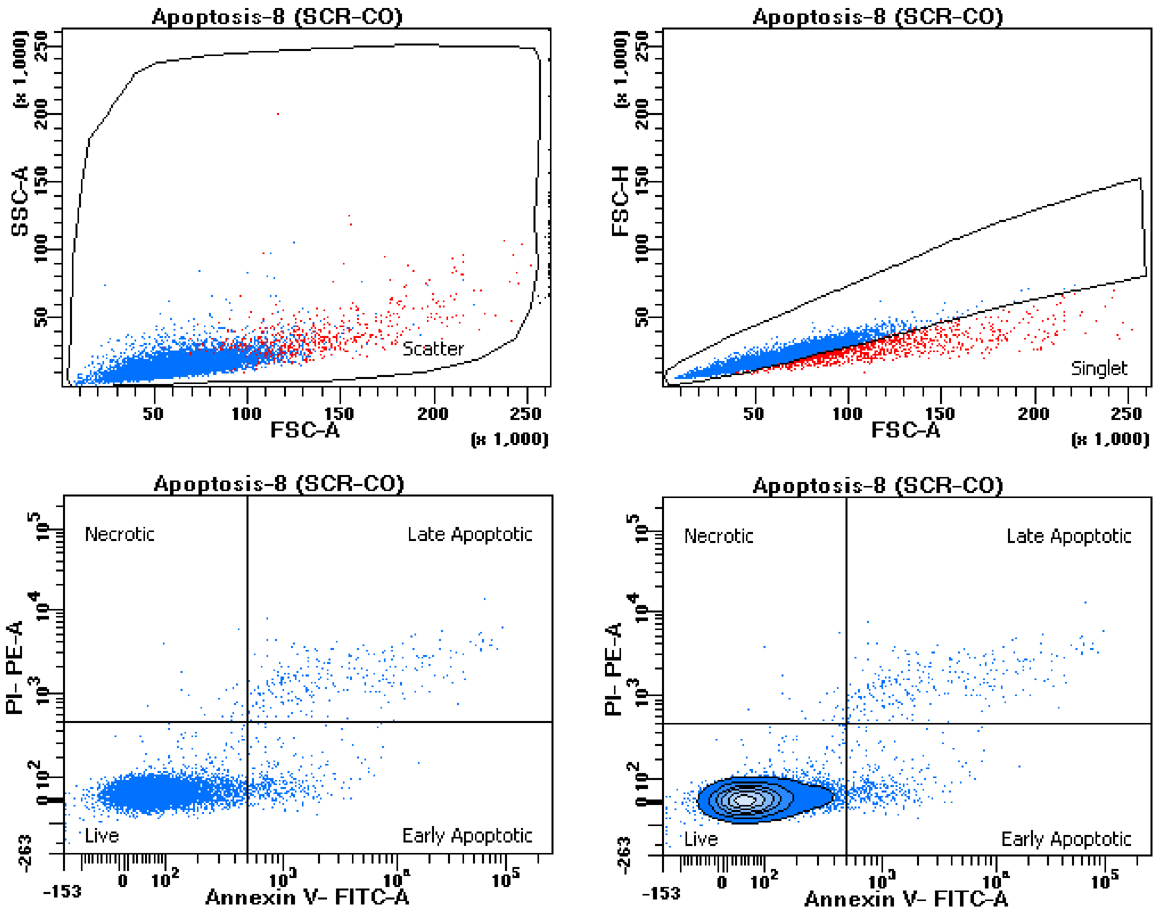

Tube: 8 (SCR-CO)

Population	#Events	%Parent	%Total
All Events	10,000	#####	100.0
Scatter	9,953	99.5	99.5
Singlet	8,560	86.0	85.6
Necrotic	26	0.3	0.3
Late Apoptotic	258	3.0	2.6
Live	7,969	93.1	79.7
Early Apoptotic	307	3.6	3.1

Experiment Name: 19Sept2019_Apoptosis
Tube Name: 8 (SCR-CO)
Record Date: Sep 19, 2019 3:32:55 PM

Population	#Events	%Parent	Annexin V- ... Median	PI- PE-A Median	Time Median
Scatter	9,953	99.5	72	31	443
Singlet	8,560	86.0	68	29	441
Necrotic	26	0.3	379	789	342
Late Apoptotic	258	3.0	2,324	1,551	413
Live	7,969	93.1	63	27	441
Early Apoptotic	307	3.6	839	59	464

***Fig.S12 (A) SSC/FSC plots** of Flow cytometry based apoptotic analysis using AnnexinV /FITC – PI staining of A549 cells treated with Co-culture Conditioned medium of H1299 cells transfected with Scrambled for 48 h (Fig.7O).*

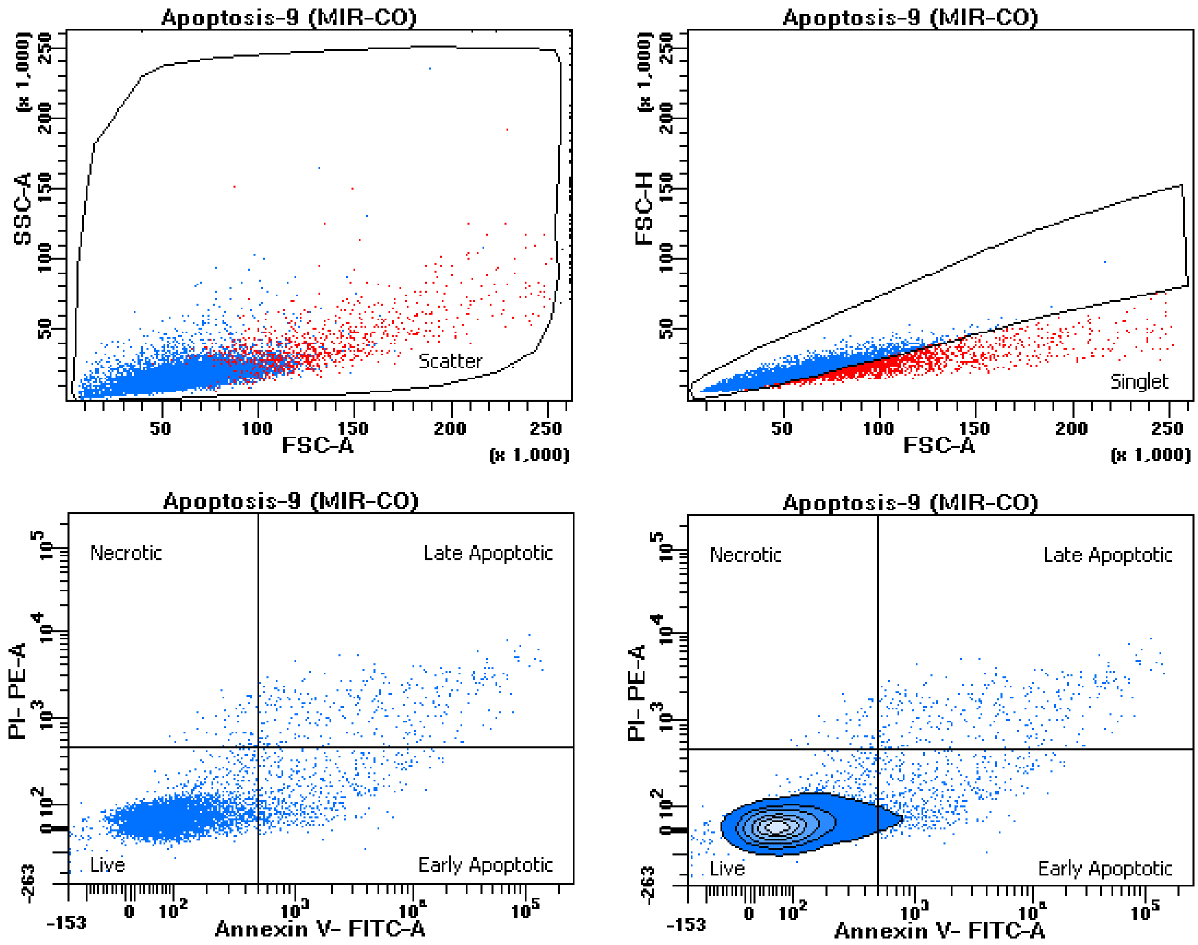

Tube: 9 (MIR-CO)

Population	#Events	%Parent	%Total
All Events	10,000	#####	100.0
Scatter	9,916	99.2	99.2
Singlet	7,318	73.8	73.2
Necrotic	51	0.7	0.5
Late Apoptotic	309	4.2	3.1
Live	6,480	88.5	64.8
Early Apoptotic	478	6.5	4.8

Experiment Name: 19Sept2019_Apoptosis
Tube Name: 9 (MIR-CO)
Record Date: Sep 19, 2019 3:33:56 PM

Population	#Events	%Parent	Annexin V- ... Median	PI- PE-A Median	Time Median
Scatter	9,916	99.2	90	43	739
Singlet	7,318	73.8	84	40	730
Necrotic	51	0.7	347	721	537
Late Apoptotic	309	4.2	4,915	1,536	753
Live	6,480	88.5	73	35	728
Early Apoptotic	478	6.5	1,138	130	760

***Fig.S12 (B) SSC/FSC plots** of Flow cytometry based apoptotic analysis using AnnexinV /FITC – PI staining of A549 cells treated with Co-culture Conditioned medium of H1299 cells transfected with miR-34a-5p mimic for 48 h (Fig.7O).*

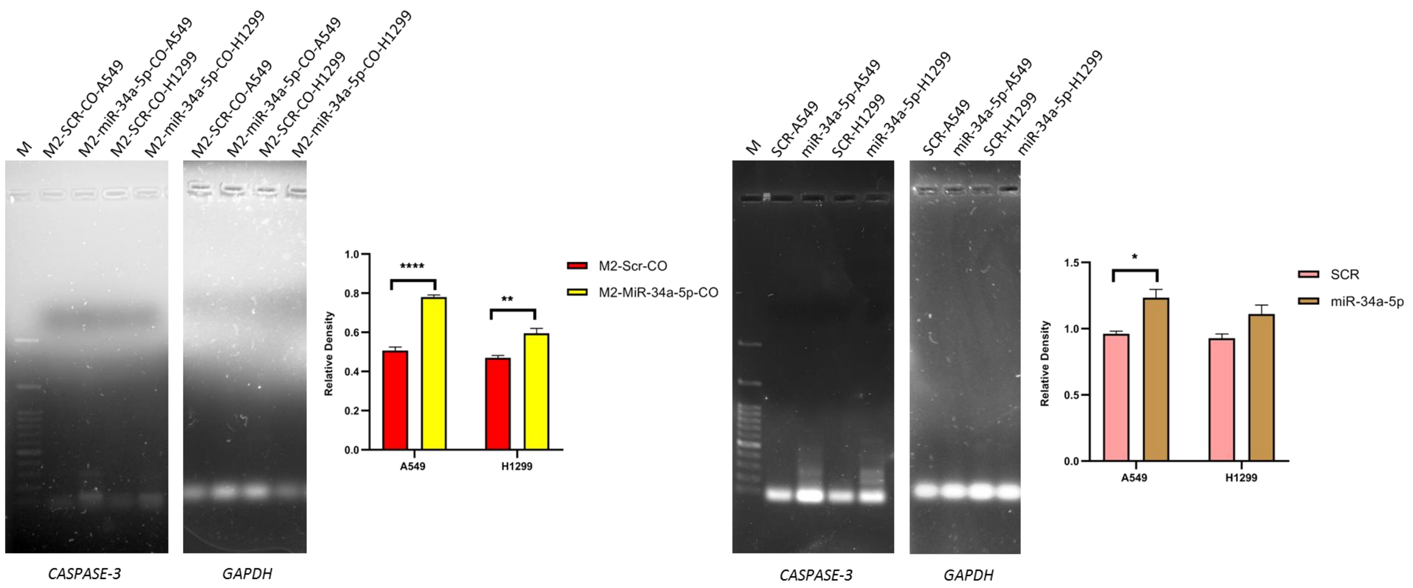

Fig.S13 Relative expression of Caspase-3 upon transfection of *(A) M2-Scr-CO and M2-miR-34a-5p-CO in A549 and H1299 cells; (B) Scrambled and miR-34a-5p in A549 and H1299 cells. The expression was determined via semi-quantitative RT-PCR and relative densities of the bands are plotted. GAPDH was used as an endogenous control. (Scr-scrambled transfection, miR-mir-34a-5p transfection).* *p < 0.05, **p < 0.01, ***p < 0.001,****p<0.0001.
