# Supplementary material for: Comprehensive Integrative Analysis Reveals the Association of KLF4 with Macrophage Infiltration and Polarization in Lung Cancer Microenvironment
Source: Cells. 2021 Aug 14;10(8):2091. doi: 10.3390/cells10082091 (PMC8392240; doi:10.3390/cells10082091)
Supplement: Supplementary file 1 [file cells-10-02091-s001.zip › cells-1312603-supplementary/SUPPLEMENTARY/SUPPLEMENTARY TABLES.pdf]

SUPPLEMENTARY TABLES

Table S1. List of Differentially Expressed Genes (DEGs) in NSCLC

Gene.symbol	adj.P.Val	logFC
FABP4	7.06E-81	-6.025674699
LOC401286	1.17E-80	-7.605771084
SH3GL3	2.28E-72	-3.55573494
LYVE1	2.53E-69	-4.650855422
FMO2	6.86E-68	-3.657638554
ITLN2	1.02E-67	-6.018506024
CLEC1A	2.43E-67	-3.160566265
AGER	6.10E-65	-5.186690763
MMRN1	8.09E-65	-4.135746988
ADH1B	8.15E-65	-4.893542169
PECAM1	1.94E-64	-2.504433735
CLEC3B	1.32E-63	-4.662951807
CAV1	2.21E-63	-4.112295181
FAM107A	4.72E-63	-5.17653012
LDB2	2.11E-62	-2.935891566
RGCC	4.06E-61	-2.769481928
TCF21	7.77E-61	-4.20548996
LGI3	2.06E-60	-3.55446988
JAM2	6.84E-60	-3.048048193
KANK3	7.23E-60	-3.095
SPOCK2	3.69E-59	-3.142542169
S1PR1	4.99E-59	-2.862518072
TEK	6.69E-59	-3.760560241
RHOJ	2.89E-58	-2.887698795
C10orf67	3.54E-58	-3.552915663
ETV4	2.49E-57	4.165246988
PLAC9	4.05E-57	-3.772301205
SOX17	5.06E-57	-3.214036145
CNRIP1	6.87E-57	-2.918253012
CYYR1	8.42E-57	-2.98539759
SGCG	8.66E-57	-2.969036145
ADAMTS8	9.35E-57	-3.490168675
GHR	1.14E-56	-3.106084337
EMCN	2.69E-56	-3.694506024
MFAP4	3.55E-56	-3.379578313
SCARA5	5.47E-56	-4.226168675
CCM2L	6.78E-56	-3.030771084
DNASE1L3	6.78E-56	-4.666686747
CA4	6.78E-56	-5.49026506
GSTM5	5.91E-55	-2.857

PTPRB	3.36E-54	-3.158433735
CDH5	3.51E-54	-2.845975904
FHL1	4.91E-54	-3.47639759
ACADL	1.01E-53	-3.369927711
COX7A1	1.72E-53	-2.598481928
GDF10	3.08E-53	-3.793975904
PI16	4.95E-53	-4.384915663
CDO1	6.20E-53	-3.342626506
GPR146	1.23E-52	-2.096036145
STX1A	1.75E-52	3.046927711
SRPX	1.88E-52	-3.267445783
CCL14	2.15E-52	-4.02786747
CD93	3.98E-52	-2.452614458
LIMS2	8.20E-52	-2.613650602
COL6A6	1.09E-51	-3.549891566
HEG1	1.53E-51	-2.27473494
GLIPR2	2.05E-51	-2.151421687
WNT3A	2.57E-51	-4.354108434
HSPA12B	5.22E-51	-2.814421687
RPL13AP17	5.82E-51	-3.61053012
MCEMP1	9.08E-51	-5.051819277
MMP11	9.20E-51	5.120180723
SPARCL1	1.16E-50	-2.713596386
FAM110D	1.47E-50	-2.887620482
SVEP1	1.53E-50	-2.898951807
LTC4S	1.79E-50	-3.53213253
FAM150B	3.16E-50	-3.626084337
FHL5	3.25E-50	-2.571987952
HIGD1B	5.54E-50	-3.63726506
HBB	8.17E-50	-4.385626506
C16orf59	9.59E-50	2.890891566
CRYAB	9.62E-50	-3.034253012
TM6SF1	1.17E-49	-2.512313253
IGSF9	1.31E-49	3.308807229
VWF	2.15E-49	-2.245951807
HBA2	2.61E-49	-4.145915663
NLRC4	3.00E-49	-2.225373494
RTKN2	1.38E-48	-3.290578313
C1orf162	1.76E-48	-2.380614458
CFD	2.30E-48	-3.03673494
CLEC14A	1.28E-47	-2.641216867
HBA1	2.57E-47	-3.943385542
TOP2A	3.01E-47	4.005566265
HSPB2	4.60E-47	-2.35573494
FGFBP2	5.02E-47	-3.41246988

ACVRL1	6.96E-47	-2.696072289
C1orf186	6.97E-47	-2.986108434
OLFML1	9.12E-47	-2.195445783
DPEP2	9.12E-47	-2.744084337
ABI3BP	9.12E-47	-2.960457831
CENPF	1.90E-46	3.320746988
SAPCD2	2.26E-46	3.406060241
SLC31A2	2.47E-46	-2.216819277
PPP1R14A	3.20E-46	-2.192903614
CCL23	3.37E-46	-2.771855422
TGFBR3	6.94E-46	-2.540831325
CPA3	8.67E-46	-3.501578313
EPAS1	9.41E-46	-2.257
MT1M	1.03E-45	-4.468048193
PTGER4	1.08E-45	-2.146349398
CD52	1.43E-45	-2.846180723
F10	2.00E-45	-2.645289157
RECQL4	2.22E-45	2.787084337
CASQ2	2.53E-45	-2.789771084
TMEM100	3.15E-45	-4.697192771
MS4A2	3.77E-45	-3.134939759
PEBP4	3.79E-45	-4.543433735
EFCC1	4.03E-45	-2.658253012
PKNOX2	4.38E-45	-2.361048193
SERTM1	8.18E-45	-2.187313253
HSPC324	1.06E-44	-3.367855422
FPR2	1.18E-44	-3.039457831
FIGF	1.53E-44	-3.379939759
FXYD6	1.89E-44	-2.250409639
CDCA8	2.12E-44	2.551650602
HOXA5	2.15E-44	-2.478963855
BCHE	2.62E-44	-3.257855422
ADH1A	4.30E-44	-4.624373494
VSIG4	6.38E-44	-3.202710843
GPT2	6.99E-44	2.480048193
SELP	7.31E-44	-2.651409639
SDPR	1.86E-43	-3.218433735
CLDN5	2.03E-43	-2.871216867
CALCRL	2.18E-43	-3.334433735
STIL	2.81E-43	2.389216867
LAMP3	3.29E-43	-2.919325301
TCEAL2	5.13E-43	-3.510891566
REM1	5.30E-43	-2.47713253
C2orf40	6.51E-43	-4.264120482
INMT	7.19E-43	-3.73286747

ARHGEF16	7.93E-43	2.294216867
GIMAP4	1.32E-42	-2.057807229
CD300C	1.39E-42	-3.018349398
TMEM88	1.52E-42	-2.520036145
GIMAP8	1.56E-42	-2.105313253
ADRB2	1.75E-42	-2.666566265
C5AR1	1.79E-42	-2.58460241
FGD5	1.91E-42	-2.398445783
MAMDC2	2.79E-42	-3.856831325
GKN2	2.94E-42	-5.438819277
ADIRF	2.98E-42	-3.043746988
C10orf54	3.26E-42	-2.211168675
SOSTDC1	3.50E-42	-3.993385542
CPED1	3.53E-42	-2.372710843
SEMA6A	3.61E-42	-2.938228916
SERINC2	3.83E-42	2.958156627
RASL12	4.86E-42	-2.252361446
VGLL3	6.20E-42	-2.764927711
SLC2A1	6.63E-42	2.939891566
SOX7	7.90E-42	-3.01883735
FAM212A	9.43E-42	-2.091445783
ANGPT1	1.04E-41	-2.946475904
IGSF10	1.04E-41	-2.376409639
RAMP3	1.21E-41	-3.011475904
RASIP1	1.51E-41	-2.37573494
GIMAP5	1.69E-41	-2.282457831
CDC20	1.81E-41	3.024084337
SRGN	1.92E-41	-2.553048193
CELSR3	2.05E-41	3.167156627
GPX3	2.58E-41	-2.180240964
DES	3.26E-41	-3.113783133
ERO1A	3.45E-41	2.049891566
PADI4	5.04E-41	-2.847493976
HOOK1	5.43E-41	2.413891566
ANOS1	5.43E-41	-3.171385542
AOC3	8.39E-41	-2.614939759
FCGR1B	8.40E-41	-2.830873494
CDH13	1.04E-40	-2.727192771
FUT2	1.75E-40	3.497903614
HCAR2	2.05E-40	-2.541578313
PROM2	2.12E-40	2.821626506
MNDA	3.62E-40	-3.284036145
GCNT3	4.08E-40	4.924301205
SASH1	5.08E-40	-2.107331326
TTK	5.09E-40	2.824746988

KANK2	5.33E-40	-2.21613253
PCOLCE2	6.26E-40	-3.690024096
HBEGF	6.48E-40	-2.515337349
LMO2	7.54E-40	-2.293301205
GPD1	9.01E-40	-2.630445783
KRT8	9.14E-40	2.006337349
FOXF1	9.68E-40	-2.955855422
HSPB6	1.05E-39	-2.697698795
ASPM	1.37E-39	3.265433735
SCN4B	1.40E-39	-2.605686747
LRRN3	1.47E-39	-2.8885
TNNC1	1.94E-39	-3.664204819
ID3	2.00E-39	-2.309891566
FIBIN	2.26E-39	-2.457072289
PMP22	2.78E-39	-2.601771084
LHFP	2.95E-39	-2.077542169
FCN1	3.97E-39	-2.962710843
WIF1	4.31E-39	-4.559108434
ROBO4	4.67E-39	-2.284710843
CMTM2	5.67E-39	-2.917879518
TLR4	6.04E-39	-2.012361446
TK1	7.40E-39	2.639156627
CCNF	8.05E-39	2.150373494
FPR1	8.06E-39	-3.420506024
ACKR1	1.08E-38	-3.682698795
CCDC178	1.24E-38	-3.193662651
SPC24	1.30E-38	2.407674699
AOX1	1.58E-38	-2.998506024
SNCA	1.74E-38	-2.636150603
CBLC	2.04E-38	2.625240964
TIMP3	2.43E-38	-2.535578313
FXYD1	2.63E-38	-2.572598394
AQP4	3.38E-38	-3.433198795
CLEC12A	4.27E-38	-2.669957831
GPC3	5.32E-38	-3.300626506
PID1	6.03E-38	-2.998337349
ITPKA	1.16E-37	4.456325301
CDCA5	1.29E-37	2.652686747
LILRA2	1.55E-37	-2.913385542
THBD	2.16E-37	-2.550204819
GPBAR1	2.33E-37	-2.201662651
CAMP	2.87E-37	-3.689614458
AURKB	3.12E-37	3.010457831
HJURP	3.42E-37	2.663192771
ANLN	3.53E-37	2.55346988

SBSPON	3.73E-37	-3.496746988
CHTF18	3.94E-37	2.343120482
RETN	3.99E-37	-3.869421687
SDHAP3	4.24E-37	2.518831325
NECTIN4	4.60E-37	2.353385542
UBE2T	4.63E-37	2.603855422
PLEK2	5.61E-37	2.49453012
NECAB1	7.30E-37	-2.373198795
FCN3	7.91E-37	-4.299174699
JAML	9.31E-37	-2.132228916
PROK2	1.05E-36	-2.760506024
MS4A7	1.13E-36	-2.631933735
GNG11	1.73E-36	-3.050891566
AQP9	1.74E-36	-2.665144578
SLC19A3	1.91E-36	-3.281253012
EFEMP1	1.92E-36	-2.586759036
ADAM8	2.18E-36	2.507156627
PLA2G4F	2.21E-36	-3.069180723
SLIT3	2.43E-36	-3.063054217
C1QTNF7	2.45E-36	-2.077662651
PRC1	3.54E-36	2.13353012
FOLR3	4.67E-36	-4.097554217
NCAPG	4.95E-36	2.612325301
RECK	5.00E-36	-2.361680723
PRELP	5.00E-36	-2.15060241
BTNL9	5.48E-36	-2.662951807
ALOX5AP	6.73E-36	-2.349301205
F12	6.80E-36	2.478795181
CPB2	7.85E-36	-3.624313253
ID2	7.92E-36	-2.174771085
GJC2	8.01E-36	-2.419156627
KIF2C	8.64E-36	2.521373494
TPX2	1.02E-35	2.769855422
ESAM	1.65E-35	-2.228072289
RNF43	1.71E-35	2.118493976
TBX4	2.11E-35	-2.106710843
COL13A1	2.11E-35	-2.313120482
TMEM132A	2.18E-35	2.271
GJA4	2.39E-35	-2.017987952
ADTRP	2.45E-35	-2.91386747
WISP2	2.49E-35	-2.896759036
TMEM204	2.92E-35	-2.012457831
ADAMTS1	2.93E-35	-2.814168675
TROAP	2.97E-35	2.626795181
HSPB8	3.24E-35	-2.724108434

OLR1	3.27E-35	-2.662084337
WNT3	3.83E-35	2.066506024
PRG4	3.94E-35	-4.063
OR7E37P	4.52E-35	-2.056433735
TMPRSS4	4.87E-35	2.980879518
CES1P1	5.01E-35	-3.705819277
POLQ	5.40E-35	2.408746988
CRTAC1	5.41E-35	-3.803036145
PTGDS	6.23E-35	-2.630240964
AURKA	7.36E-35	2.397885542
AGTR1	7.38E-35	-2.494319278
CAV2	8.13E-35	-2.979783133
CD33	8.20E-35	-2.610783133
ADAMTSL3	8.65E-35	-2.291915663
CKAP2L	8.85E-35	2.599843373
CDC45	8.99E-35	2.638337349
IL6	1.01E-34	-3.94139759
RAMP2	1.12E-34	-2.099843374
HBG2	1.24E-34	-3.643325301
SOCS2	1.26E-34	-2.623036145
SALL4	1.29E-34	3.018674699
COL10A1	1.44E-34	3.48446988
KIAA1324L	1.50E-34	-2.679048193
ARHGAP31	1.57E-34	-2.095710843
EGR2	1.96E-34	-2.274096386
TNFSF13	2.49E-34	-2.219951808
FBLN5	2.75E-34	-2.223379519
SLIT2	3.47E-34	-2.067060241
CDT1	3.70E-34	2.61513253
HSD11B1	4.23E-34	-2.397236948
TMOD1	5.88E-34	-2.54886747
GRASP	6.75E-34	-2.173289157
FCER1G	7.04E-34	-2.042759036
SMAD6	7.09E-34	-2.811795181
KIF20A	8.61E-34	2.595831325
HSPB3	8.90E-34	-2.263554217
CD69	9.95E-34	-2.709849398
TMEM45B	1.06E-33	2.796337349
AUNIP	1.15E-33	2.051156627
MARCO	1.33E-33	-3.664638554
C7	1.45E-33	-3.42660241
CLIC5	1.47E-33	-3.141771084
PLSCR4	1.74E-33	-2.045855422
TYROBP	2.07E-33	-2.187168675
PHLDA2	2.13E-33	2.711903614

TUBB3	3.38E-33	3.395493976
ANKRD1	3.82E-33	-3.539566265
NCF1	3.98E-33	-3.43653012
SPTBN2	4.60E-33	2.026433735
C1QA	5.21E-33	-2.307722892
MAOB	5.85E-33	-2.432759036
FMO3	6.72E-33	-2.49123494
COLEC12	7.12E-33	-2.483891566
STXBP6	7.20E-33	-3.101072289
GPER1	1.13E-32	-2.102722892
CD300LF	1.52E-32	-2.471807229
GINS2	1.66E-32	2.285036145
PDK4	2.06E-32	-3.018975904
C1orf106	2.15E-32	2.53939759
KCNA5	2.32E-32	-2.217506024
FGF11	2.71E-32	2.495012048
CERCAM	2.73E-32	2.193325301
LRRC36	3.10E-32	-2.798698795
CCNB1	3.15E-32	2.439843373
CCNB2	3.22E-32	2.627746988
ENPP2	3.31E-32	-2.146343374
DBNDD1	3.79E-32	2.105903615
PDE2A	3.91E-32	-2.014927711
C8B	4.40E-32	-2.956253012
COL1A1	4.47E-32	2.859903614
S100A4	4.61E-32	-2.318548193
LPL	4.75E-32	-2.880783133
COX4I2	5.73E-32	-2.364746988
ADGRE1	5.95E-32	-2.023554217
SFN	7.35E-32	2.60860241
TMEM74B	8.12E-32	-2.33626506
MELK	9.40E-32	2.80063253
CCNA2	1.03E-31	2.238084337
CEP55	1.16E-31	2.28746988
HBG1	1.23E-31	-3.270807229
BTG2	1.27E-31	-2.154421687
FUT3	1.35E-31	2.758168675
CRABP2	1.62E-31	3.748759036
RFTN2	2.43E-31	-2.051674699
LILRA5	2.79E-31	-2.368722892
FOXM1	3.40E-31	2.520198796
KIF15	3.66E-31	2.113987952
DLGAP5	4.13E-31	2.149180723
ARHGAP44	4.19E-31	-2.063409639
WASF3	4.30E-31	-2.336855422

DNAJC22	4.90E-31	2.627240964
SPDEF	6.77E-31	3.551216867
RAB26	7.56E-31	2.291216867
ALDH1A2	7.67E-31	-2.518722892
RHOD	9.31E-31	2.028156627
ECM2	9.40E-31	-2.025042169
FAM189A2	9.80E-31	-2.638638554
SLC6A4	1.14E-30	-3.801036145
KIF11	1.25E-30	2.139578313
HOXA4	1.39E-30	-2.076939759
KIFC1	1.48E-30	2.45039759
MGP	1.57E-30	-2.076090362
ZFP36	1.75E-30	-2.02926506
OSR1	2.04E-30	-2.351277108
WFDC1	2.20E-30	-2.576168675
GATA2	2.33E-30	-2.324879518
SOX18	3.03E-30	-2.55126506
CCDC89	3.77E-30	-2.080554217
METTL7B	4.05E-30	3.185542169
S100A8	4.32E-30	-3.043554217
GPA33	4.83E-30	-2.576566265
MARVELD3	6.27E-30	2.03151004
SLC22A18AS	7.67E-30	2.62446988
XDH	8.61E-30	2.450373494
ITLN1	1.10E-29	-5.118361446
ECT2	1.25E-29	2.00953012
CNN1	1.35E-29	-2.340120482
HMMR	1.53E-29	2.493120482
RGS17	1.72E-29	3.259879518
CST1	2.01E-29	4.753855422
GIMAP1	2.42E-29	-2.055939759
AADAC	3.94E-29	-3.180180723
MYH4	4.49E-29	-2.136518072
KIF4A	4.87E-29	2.259566265
TMEM178A	4.88E-29	-2.33486747
DPT	5.44E-29	-2.638301205
HSD17B6	5.52E-29	-3.27353012
NPNT	6.90E-29	-2.288301205
SH3GL2	7.15E-29	-2.570710843
PODXL2	9.48E-29	2.940012048
KIF26B	9.62E-29	2.136698795
GOLGA7B	1.02E-28	2.616518072
PLA2G1B	1.16E-28	-4.083975904
CDCA3	1.24E-28	2.384481928
LEFTY2	1.26E-28	-2.234915663

GLDN	1.56E-28	-2.943108434
CYP4B1	2.21E-28	-4.088295181
CXCR2	2.22E-28	-2.165771084
ASF1B	2.48E-28	2.192240964
PLEKHG6	3.16E-28	2.058096386
C17orf53	4.21E-28	2.230385542
IL1B	5.01E-28	-2.672228916
CD163	5.15E-28	-2.155028112
VIPR1	5.50E-28	-2.175927711
TSPAN7	7.10E-28	-3.315674699
TUBB6	9.20E-28	-2.245759037
NCF2	1.01E-27	-2.040313253
UNC5CL	1.13E-27	2.071156627
GPM6B	1.46E-27	-2.853257028
PDE7B	1.56E-27	-2.104445783
HMGB3	1.94E-27	2.767036145
EEF1A2	2.03E-27	5.045012048
DUOXA1	2.12E-27	-2.566951807
FERMT1	2.21E-27	2.414289157
SPP1	2.98E-27	3.844530121
NIM1K	3.06E-27	-2.277771084
MAL	3.29E-27	-2.295957831
KCNK5	3.64E-27	2.039048193
RALGPS2	3.73E-27	2.156626506
MSRB3	5.63E-27	-2.466799197
BUB1	5.76E-27	2.246578313
CTSG	7.53E-27	-3.22853012
GJB2	7.95E-27	3.035036145
ATP10B	8.61E-27	3.259361446
F13A1	1.09E-26	-2.504975904
NOSTRIN	1.22E-26	-2.245445783
CXCL2	1.26E-26	-2.875686747
GSTM3	1.52E-26	-2.287349398
CD36	1.55E-26	-3.57313253
CA2	2.19E-26	-2.558319277
TRIM46	2.21E-26	2.009
ESPN	2.59E-26	2.625987952
CNFN	2.65E-26	2.152506024
CACNA2D2	2.95E-26	-2.45960241
TPPP	2.97E-26	-2.050337349
SIX1	3.75E-26	2.345337349
ZBED2	4.03E-26	-2.83826506
CYTL1	4.30E-26	-2.390277108
STK32A	4.70E-26	2.415686747
FCER1A	5.02E-26	-3.025409639

MS4A15	6.04E-26	-3.273108434
CDH3	6.05E-26	2.825072289
GLB1L2	6.90E-26	2.544506024
FAM64A	7.46E-26	2.074240964
LCN6	8.04E-26	-2.064650602
PLXNB3	1.62E-25	2.033554217
CXCL12	1.69E-25	-2.707084338
NMU	1.81E-25	3.283096386
AQP1	1.83E-25	-2.858783133
TPSAB1	1.88E-25	-2.525686747
SPAG4	2.49E-25	2.139277108
ACTG2	2.50E-25	-2.115144578
SIX4	3.07E-25	2.247674699
GAS1	3.86E-25	-2.451066266
VEPH1	4.80E-25	-2.127036145
CAPN12	5.52E-25	2.368855422
DAPK2	7.38E-25	-2.012915663
CYP27B1	1.10E-24	2.144180723
S100A3	1.13E-24	-2.668373494
DEFA1	1.15E-24	-2.938572289
DEFA1B	2.21E-24	-2.263108434
ADARB1	2.96E-24	-2.046722892
REEP1	4.13E-24	-2.338915663
CLC	4.50E-24	-2.450313253
KRT4	5.89E-24	-2.451253012
MUC20	6.53E-24	2.413554217
PTGES	6.66E-24	2.10060241
MMP9	8.81E-24	2.69673494
BMP6	9.49E-24	-2.178590361
FAM181A	1.19E-23	-2.536771084
MYH11	1.28E-23	-2.35776506
S100A12	1.31E-23	-2.08913253
FOSB	1.53E-23	-3.385204819
ITM2A	1.81E-23	-2.067861446
PLA2G3	2.01E-23	-2.142373494
PITX1	2.41E-23	3.919144578
CCL2	2.79E-23	-2.010289157
COLGALT2	2.98E-23	-2.194072289
APLN	4.65E-23	-2.014518072
ANXA3	7.43E-23	-2.464325301
COMP	8.68E-23	3.730554217
NRIP3	1.01E-22	2.077060241
PGC	1.49E-22	-4.334975904
SFTPC	1.76E-22	-4.338289157
CDKN3	2.97E-22	2.086746988

PBK	3.00E-22	2.085192771
ABCA3	4.79E-22	-2.145289157
SFRP1	1.26E-21	-2.589216867
FRAS1	1.66E-21	-2.492927711
XKRX	2.17E-21	2.553240964
LYZ	2.18E-21	-2.875963856
DEFA3	2.79E-21	-2.328060241
GPR37	3.00E-21	2.556108434
CA9	4.32E-21	2.879445783
RERG	4.82E-21	-2.009759036
SEMA5A	5.60E-21	-2.191548193
PLAC8	6.23E-21	-2.390608434
PTPRH	7.55E-21	2.07046988
KISS1R	8.81E-21	2.800746988
CCNO	1.08E-20	2.02313253
GOLGA8A	1.23E-20	2.293289157
SFTPD	1.46E-20	-2.583409639
CCL3	1.56E-20	-2.315710843
GATA6-AS1	1.59E-20	-2.144313253
CTHRC1	1.77E-20	2.276096386
SLC1A1	2.04E-20	-2.17786747
ALG1L	2.39E-20	2.562596386
FAM167A	3.08E-20	-2.210144578
SLPI	3.24E-20	-2.457903614
LRRK2	3.72E-20	-2.12846988
SPINK1	5.08E-20	4.416626506
SUSD2	6.46E-20	-2.189855422
FAM178B	1.11E-19	2.081072289
CLPSL2	2.94E-19	2.242048193
BCO1	3.75E-19	2.077614458
TMSB15A	4.40E-19	-2.453457831
BIRC5	4.68E-19	2.36386747
KCNK12	6.32E-19	2.331506024
ARSE	1.48E-18	2.047108434
ZNF385B	2.80E-18	-2.709903614
MBTD1	3.28E-18	2.515493976
ROPN1L	5.56E-18	-2.60786747
IGFBP6	7.80E-18	-2.021409639
ALPL	1.16E-17	-2.170759036
SLN	1.19E-17	-2.240253012
FAM216B	1.39E-17	-2.954975904
GALNT3	1.44E-17	2.073939759
SYT12	1.86E-17	2.113951807
C11orf88	2.09E-17	-2.943192771
KRT80	2.42E-17	2.103843374

SCGB1A1	2.59E-17	-4.57286747
CP	2.87E-17	2.332120482
P3H2	4.97E-17	-2.028060241
C6	5.17E-17	-2.431506024
FOXA3	6.28E-17	2.257373494
MT1E	1.12E-16	-2.011632531
STRA6	1.30E-16	2.116927711
ARMCX1	1.41E-16	-2.034771084
FOXP1-IT1	1.75E-16	2.01039759
ALDH1A1	1.82E-16	-2.346710844
SSTR2	2.21E-16	2.15613253
SCG5	2.68E-16	2.50886747
VSTM2L	2.74E-16	2.396325301
SFTPA1	3.14E-16	-3.955987952
LMOD3	3.29E-16	2.085975904
EFCAB1	3.72E-16	-2.719108434
BAIAP2L2	3.73E-16	2.317120482
CEACAM5	6.33E-16	3.433506024
MAP7D2	7.17E-16	2.044626506
NEK2	7.70E-16	2.12713253
DNAJC12	9.93E-16	2.350542169
MORN5	1.24E-15	-2.184144578
CXCL5	2.38E-15	-2.107945783
CH25H	2.51E-15	-2.157614458
VSIG2	2.81E-15	-2.467506024
CFAP77	2.95E-15	-2.15313253
C22orf15	3.91E-15	-2.049518072
MMP1	4.55E-15	2.216313253
LOC729860	4.68E-15	2.038216867
C20orf85	6.66E-15	-2.847819277
LEMD1	8.83E-15	2.042361446
TMPRSS6	9.06E-15	2.186385542
ITCH-IT1	1.48E-14	2.254879518
MSR1	3.08E-14	-2.168289156
PLA2G2A	3.53E-14	-2.052060241
PSG1	3.63E-14	2.411614458
SLC16A9	5.34E-14	2.014626506
TEKT1	6.09E-14	-2.518795181
IGF2BP3	6.46E-14	2.273686747
SCGB3A2	7.39E-14	-2.535012048
HABP2	1.05E-13	2.377072289
B3GNT6	3.62E-13	2.157313253
TMEM190	4.42E-13	-2.147036145
ABCA4	5.27E-13	2.226795181
ZBTB16	5.33E-13	-2.137594377

CYP24A1	5.37E-13	2.373879518
CXCL13	6.03E-13	2.065746988
SLC39A8	6.75E-13	-2.308819277
DNAH2	1.30E-12	2.088939759
WDR66	1.79E-12	2.62639759
PCP4	2.12E-12	2.835445783
MUC21	2.54E-12	2.012885542
KLF4	4.26E-12	2.933710844
MUC16	6.80E-12	2.48846988
MMP12	8.35E-12	2.41283735
C4BPA	8.90E-12	-2.134024096
KRT15	1.63E-11	2.114216867
BEX1	1.69E-11	-2.035355422
MYEOV	4.46E-11	2.036903614
SHISA2	8.26E-11	-2.133903614
ABCA8	8.85E-11	-2.12736747
CDHR3	2.22E-10	-2.131542169
UBE2C	3.11E-10	2.219493976
AOC1	5.67E-10	2.03546988
HP	9.95E-10	-2.124048193
GPX2	1.15E-09	2.706493976
FAM83A	1.28E-09	3.353305221
MS4A8	1.58E-09	-2.218759036
KIF1A	1.59E-09	2.247650602
TM4SF4	4.14E-09	2.347807229
AKR1B10	6.16E-09	2.326626506
SCGB3A1	8.00E-09	-2.10573494
TCN1	3.19E-08	2.385240964
CRLF1	6.25E-08	2.02239759
PAEP	2.37E-07	2.05783735
DCN	2.17E-06	-2.561927711
PGM5	3.62E-06	-2.06606747

Table S2. List of Differentially Expressed miRNAs (DEMs) in NSCLC

miRNA_ID	adj.P.Val	logFC
hsa-miR-135a-3p	5.08E-22	0.512769973
hsa-miR-4301	1.19E-20	0.299462336
hsa-miR-4721	1.23E-19	0.727174851
hsa-miR-1306-3p	1.26E-19	0.344086877
hsa-miR-4465	2.21E-19	0.658898648
hsa-miR-320a	6.16E-19	0.484948304
hsa-miR-296-5p	5.56E-18	-0.837156692
hsa-miR-3935	8.61E-18	0.305621515

hsa-miR-5189	2.16E-17	0.517632038
hsa-miR-5580-3p	2.89E-17	0.238891697
hsa-miR-4430	4.49E-17	0.486193025
hsa-miR-302f	4.71E-17	-0.24804573
hsa-miR-4793-5p	4.71E-17	0.409991705
hsa-miR-4442	1.48E-16	0.555100877
hsa-miR-3180-3p	1.52E-16	-0.729492197
hsa-miR-4653-3p	6.00E-16	0.662450899
hsa-miR-4499	3.80E-15	0.473188139
hsa-miR-3972	1.03E-14	-0.549521473
hsa-miR-4496	1.45E-14	0.439847873
hsa-miR-602	1.58E-14	0.342729443
hsa-miR-4769-3p	1.58E-14	-0.620769774
hsa-miR-1228-3p	1.86E-14	-0.56267776
hsa-miR-1237	1.86E-14	-0.726393416
hsa-miR-4476	1.97E-14	0.589765543
hsa-miR-542-5p	3.52E-14	0.407148887
hsa-miR-889	3.73E-14	0.306985536
hsa-miR-1243	4.15E-14	0.180831486
hsa-miR-4648	4.56E-14	0.318277584
hsa-miR-3679-3p	5.29E-14	-0.517127424
hsa-miR-4293	6.72E-14	0.141527777
hsa-miR-4514	1.03E-13	0.234426281
hsa-miR-5009-5p	1.04E-13	0.543471198
hsa-miR-4647	1.22E-13	0.329988182
hsa-miR-3934	1.37E-13	0.538122337
hsa-miR-642a-3p	1.98E-13	0.404619871
hsa-miR-4635	2.74E-13	0.42920817
hsa-miR-1266	5.68E-13	0.202080053
hsa-miR-4773	1.11E-12	0.228269243
hsa-miR-4462	1.11E-12	0.434025036
hsa-miR-564	1.32E-12	0.238087819
hsa-miR-4487	1.32E-12	0.290286376
hsa-miR-4749-3p	1.32E-12	-0.673482122
hsa-miR-548w	1.53E-12	-0.177861742
hsa-miR-1225-3p	1.53E-12	0.399043884
hsa-miR-4470	1.72E-12	0.461355184
hsa-miR-125b-5p	1.89E-12	-0.544394925
hsa-miR-3907	2.41E-12	0.556636286
hsa-miR-3648	3.34E-12	0.368826879
hsa-miR-891a	3.42E-12	0.269049002
hsa-miR-3676-3p	3.50E-12	-0.58244119
hsa-miR-1910	4.09E-12	0.191494282
hsa-miR-3663-3p	4.67E-12	0.446301706
hsa-miR-4535	7.32E-12	0.256901866

hsa-miR-4800-5p	7.39E-12	0.443083534
hsa-miR-3917	7.44E-12	0.34018405
hsa-miR-3622b-3p	9.42E-12	0.159900356
hsa-miR-1249	9.80E-12	-0.66020537
hsa-miR-34c-3p	1.27E-11	0.33374588
hsa-miR-4477a	1.33E-11	0.184027055
hsa-miR-3188	1.42E-11	0.602165107
hsa-miR-1305	1.56E-11	0.402410165
hsa-miR-4655-5p	1.61E-11	0.371894104
hsa-miR-3911	1.75E-11	0.417463495
hsa-miR-4478	1.91E-11	0.358794403
hsa-miR-940	1.91E-11	-0.564929338
hsa-miR-1268b	1.99E-11	0.417103868
hsa-miR-100-5p	1.99E-11	-0.529599592
hsa-miR-4634	2.24E-11	0.46377103
hsa-miR-4277	3.75E-11	-0.147983998
hsa-miR-4475	3.89E-11	0.329428017
hsa-miR-4274	4.58E-11	-0.638206766
hsa-miR-1234	4.68E-11	-0.478850382
hsa-miR-4793-3p	5.53E-11	0.374523974
hsa-miR-4743	6.97E-11	0.343098461
hsa-miR-1471	9.07E-11	0.245222678
hsa-miR-4435	9.89E-11	0.145904596
hsa-miR-4640-5p	1.13E-10	0.386486923
hsa-miR-2392	1.15E-10	0.464158223
hsa-miR-1224-3p	1.15E-10	-0.472806809
hsa-miR-3659	1.34E-10	0.254015541
hsa-miR-4460	1.39E-10	-0.212063913
hsa-miR-5190	1.39E-10	0.312441956
hsa-miR-1538	1.46E-10	-0.590298992
hsa-let-7d-5p	1.81E-10	-0.650869716
hsa-miR-4664-3p	1.87E-10	-0.536657157
hsa-miR-890	1.88E-10	0.167440841
hsa-miR-4746-3p	2.36E-10	0.354410299
hsa-let-7a-5p	2.38E-10	-0.784227989
hsa-miR-548f	2.41E-10	-0.157487902
hsa-miR-382-3p	2.41E-10	-0.296048696
hsa-miR-574-3p	2.74E-10	0.200717737
hsa-miR-550a-5p	2.98E-10	0.341344454
hsa-miR-4795-3p	3.03E-10	-0.191879904
hsa-miR-525-5p	3.12E-10	-0.348268321
hsa-miR-761	3.14E-10	-0.262426903
hsa-miR-3614-5p	3.95E-10	-0.463095986
hsa-miR-4304	4.28E-10	0.151422659
hsa-miR-4433-5p	4.35E-10	-0.528755464

hsa-miR-4507	4.84E-10	0.387697009
hsa-miR-3666	5.07E-10	0.182133724
hsa-let-7f-1-3p	5.50E-10	-0.463644056
hsa-let-7f-5p	7.83E-10	-0.525687484
hsa-miR-302e	8.27E-10	-0.16910391
hsa-miR-3944-5p	9.07E-10	0.384057464
hsa-miR-4669	9.30E-10	0.344711634
hsa-miR-5095	1.12E-09	0.454827951
hsa-miR-4757-3p	1.30E-09	-0.134363677
hsa-miR-4298	1.31E-09	0.424052056
hsa-miR-486-5p	1.31E-09	-0.606467807
hsa-miR-3938	1.37E-09	0.102400447
hsa-miR-3615	1.53E-09	0.161149372
hsa-miR-5684	1.53E-09	0.351163823
hsa-miR-3925-5p	1.56E-09	0.293880997
hsa-miR-2276	1.56E-09	0.362908642
hsa-miR-3654	1.84E-09	0.231126438
hsa-miR-1469	2.30E-09	0.352780865
hsa-miR-4787-3p	2.30E-09	-0.436357634
hsa-miR-1224-5p	2.43E-09	-0.58688235
hsa-miR-5010-3p	2.43E-09	-0.745889279
hsa-miR-593-5p	2.74E-09	0.345561272
hsa-miR-766-3p	2.93E-09	-0.594414646
hsa-miR-1208	2.93E-09	0.373692476
hsa-miR-5699	3.09E-09	0.217514742
hsa-let-7e-3p	3.25E-09	-0.255373604
hsa-miR-4665-3p	3.87E-09	-0.572606176
hsa-miR-99a-5p	3.97E-09	-0.435827897
hsa-miR-1238	3.97E-09	-0.471601908
hsa-miR-4728-3p	4.01E-09	-0.550138318
hsa-miR-134	4.02E-09	0.490419397
hsa-miR-3065-5p	4.20E-09	-0.158747844
hsa-miR-629-3p	4.32E-09	0.113922467
hsa-miR-320b	4.47E-09	0.493301421
hsa-miR-526b-5p	5.12E-09	0.609904683
hsa-miR-548e	5.21E-09	-0.160535861
hsa-miR-4661-5p	5.64E-09	-0.149462685
hsa-miR-4667-3p	5.64E-09	-0.58834733
hsa-miR-3918	5.85E-09	0.312804655
hsa-miR-133a	6.11E-09	-0.358742435
hsa-miR-4751	6.52E-09	0.191511152
hsa-miR-3937	6.52E-09	0.525856727
hsa-miR-1307-3p	6.57E-09	0.287113352
hsa-miR-4536-5p	7.19E-09	0.242514208
hsa-miR-324-3p	7.22E-09	-0.367923932

hsa-miR-4795-5p	8.62E-09	-0.213468412
hsa-miR-4753-5p	8.63E-09	0.437967785
hsa-miR-3137	8.72E-09	0.302890719
hsa-miR-191-5p	8.90E-09	-0.376294395
hsa-miR-3121-3p	1.14E-08	0.315177243
hsa-miR-3929	1.20E-08	-0.142427548
hsa-miR-3162-3p	1.51E-08	-0.532310685
hsa-miR-4799-5p	1.64E-08	-0.252524446
hsa-miR-575	1.69E-08	0.516608461
hsa-miR-4788	1.70E-08	0.40244077
hsa-miR-4494	2.03E-08	0.182850906
hsa-miR-4429	2.36E-08	0.278002251
hsa-miR-1181	2.42E-08	0.385628009
hsa-miR-26b-3p	2.48E-08	-0.21965348
hsa-miR-4716-5p	2.48E-08	-0.625680012
hsa-miR-548aw	3.05E-08	-0.178875994
hsa-miR-2116-3p	3.07E-08	-0.459194056
hsa-miR-3679-5p	3.49E-08	0.30113208
hsa-miR-3944-3p	3.55E-08	-0.310497687
hsa-miR-4486	3.66E-08	0.346693496
hsa-miR-3199	3.77E-08	0.168544964
hsa-miR-4772-5p	3.99E-08	-0.114441116
hsa-miR-4735-5p	4.05E-08	-0.141023627
hsa-miR-3074-3p	4.37E-08	0.227840873
hsa-miR-1227	4.46E-08	-0.503686299
hsa-miR-197-3p	4.68E-08	-0.132032082
hsa-let-7b-5p	4.93E-08	-0.719254544
hsa-miR-484	5.27E-08	-0.402136813
hsa-miR-378b	7.30E-08	0.247910386
hsa-miR-4459	7.68E-08	0.338111744
hsa-miR-4734	7.68E-08	0.340950893
hsa-miR-4538	7.74E-08	0.348204531
hsa-miR-4643	7.90E-08	0.14913525
hsa-miR-4713-3p	8.32E-08	0.217660029
hsa-miR-3197	8.51E-08	0.304424983
hsa-miR-4766-3p	8.86E-08	-0.115457803
hsa-miR-4526	1.01E-07	0.188952243
hsa-miR-4468	1.01E-07	0.299589178
hsa-miR-1281	1.06E-07	-0.386745904
hsa-miR-1282	1.08E-07	-0.161065003
hsa-miR-3184-3p	1.08E-07	-0.45390113
hsa-miR-4473	1.18E-07	-0.108648589
hsa-miR-2681-5p	1.18E-07	0.201270156
hsa-miR-4497	1.22E-07	0.257784979
hsa-miR-3605-5p	1.30E-07	0.218766275

hsa-miR-4513	1.33E-07	0.351283598
hsa-miR-1291	1.39E-07	0.175832195
hsa-miR-4728-5p	1.55E-07	0.443316521
hsa-miR-519a-3p	1.74E-07	-0.167257477
hsa-miR-3660	1.75E-07	0.209334091
hsa-miR-143-5p	1.85E-07	-0.160135176
hsa-miR-1294	1.93E-07	-0.113984618
hsa-miR-3973	1.96E-07	0.150523362
hsa-miR-4519	1.98E-07	0.132603425
hsa-miR-4436a	1.98E-07	0.225318434
hsa-miR-126-3p	2.00E-07	-0.668668496
hsa-miR-146a-5p	2.13E-07	-0.332925691
hsa-miR-1825	2.19E-07	-0.301386676
hsa-miR-301a-3p	2.20E-07	-0.123548672
hsa-miR-1295a	2.40E-07	0.127135658
hsa-miR-1286	2.43E-07	0.140637808
hsa-miR-656	2.48E-07	-0.166961847
hsa-miR-5195-3p	2.48E-07	0.413150444
hsa-miR-4738-3p	2.49E-07	0.293063325
hsa-miR-4489	2.54E-07	0.177654985
hsa-miR-676-3p	2.71E-07	-0.124118351
hsa-miR-557	2.75E-07	-0.436390872
hsa-miR-548at-5p	2.76E-07	-0.146167923
hsa-miR-3190-5p	2.76E-07	-0.5430664
hsa-miR-3689f	2.81E-07	0.121156381
hsa-miR-631	2.93E-07	0.130339419
hsa-miR-181b-3p	2.98E-07	-0.114952422
hsa-miR-1470	3.01E-07	-0.402821477
hsa-miR-3678-3p	3.18E-07	0.258667763
hsa-miR-4664-5p	3.30E-07	-0.568712819
hsa-miR-3177-3p	3.39E-07	0.257484641
hsa-miR-149-5p	3.44E-07	-0.114457143
hsa-miR-4529-5p	3.56E-07	0.178984631
hsa-miR-211-3p	3.66E-07	0.428935719
hsa-let-7g-5p	4.09E-07	-0.43107786
hsa-miR-3691-3p	4.26E-07	-0.106475412
hsa-miR-5708	4.26E-07	0.127023308
hsa-miR-3682-3p	4.26E-07	0.190728465
hsa-miR-3184-5p	4.38E-07	-0.571276834
hsa-miR-4445-3p	4.50E-07	0.15568628
hsa-miR-664-3p	4.50E-07	-0.519083619
hsa-miR-4530	4.53E-07	0.298985824
hsa-miR-548q	4.99E-07	0.565848338
hsa-miR-3150b-5p	5.13E-07	-0.380865114
hsa-miR-4711-3p	5.64E-07	-0.125797455

hsa-miR-4708-3p	5.64E-07	0.271509982
hsa-miR-379-5p	6.05E-07	0.156126669
hsa-miR-509-3-5p	6.05E-07	0.231875645
hsa-miR-4699-3p	6.16E-07	-0.23435197
hsa-miR-218-2-3p	6.26E-07	0.231215383
hsa-miR-4638-3p	6.95E-07	0.148265136
hsa-miR-30d-5p	7.95E-07	-0.587362376
hsa-miR-5096	8.16E-07	0.233578199
hsa-miR-5581-5p	8.61E-07	0.269321188
hsa-miR-4649-3p	8.71E-07	-0.496060056
hsa-miR-3138	8.81E-07	0.255751782
hsa-miR-4518	9.78E-07	0.191101253
hsa-miR-3120-5p	1.03E-06	-0.113580957
hsa-miR-3920	1.04E-06	-0.109419213
hsa-miR-4800-3p	1.04E-06	0.14872709
hsa-miR-423-3p	1.08E-06	-0.338646558
hsa-miR-548ao-3p	1.12E-06	0.228560006
hsa-miR-3620	1.22E-06	0.209575906
hsa-miR-4646-5p	1.29E-06	0.306317659
hsa-miR-4723-3p	1.33E-06	-0.489665583
hsa-miR-1909-5p	1.38E-06	0.23586569
hsa-miR-199b-5p	1.45E-06	-0.180446675
hsa-miR-4505	1.46E-06	0.400731172
hsa-miR-5000-5p	1.50E-06	-0.139696468
hsa-miR-4312	1.50E-06	-0.469596869
hsa-miR-4637	1.53E-06	0.231903518
hsa-miR-452-5p	1.54E-06	0.200528603
hsa-miR-4758-3p	1.56E-06	-0.415843015
hsa-miR-4445-5p	1.57E-06	0.215694609
hsa-miR-2114-5p	1.59E-06	-0.122876875
hsa-miR-3163	1.61E-06	0.116800259
hsa-miR-1292	1.67E-06	0.263567244
hsa-miR-10a-5p	1.81E-06	-0.212930086
hsa-miR-1236	1.81E-06	-0.384518687
hsa-miR-193b-5p	1.84E-06	0.22568324
hsa-miR-409-3p	1.92E-06	-0.421586389
hsa-miR-3194-3p	1.95E-06	-0.097815766
hsa-miR-1468	1.95E-06	-0.1106353
hsa-miR-4258	2.10E-06	-0.320526562
hsa-miR-184	2.13E-06	0.20557653
hsa-miR-5194	2.15E-06	0.191669177
hsa-miR-449b-3p	2.20E-06	-0.090051243
hsa-miR-625-3p	2.24E-06	-0.554700646
hsa-miR-5703	2.30E-06	0.282170626
hsa-miR-4425	2.30E-06	0.34398923

hsa-miR-3160-5p	2.32E-06	-0.158997396
hsa-miR-4327	2.34E-06	0.430130391
hsa-miR-3140-5p	2.40E-06	-0.085664207
hsa-miR-3926	2.43E-06	0.3021918
hsa-miR-324-5p	2.46E-06	-0.286540872
hsa-miR-675-5p	2.48E-06	-0.569398727
hsa-miR-34a-5p	2.53E-06	-0.289706177
hsa-miR-365a-3p	2.70E-06	-0.417352262
hsa-miR-342-3p	2.85E-06	-0.237557441
hsa-miR-298	2.98E-06	-0.354007645
hsa-miR-1324	3.06E-06	-0.095130881
hsa-miR-3118	3.12E-06	-0.102755992
hsa-miR-4275	3.19E-06	-0.117601334
hsa-miR-4677-5p	3.22E-06	-0.182426967
hsa-miR-5587-3p	3.26E-06	-0.356567178
hsa-miR-3662	3.30E-06	0.101001805
hsa-miR-1247-5p	3.30E-06	-0.344648643
hsa-miR-3646	3.51E-06	0.197992651
hsa-miR-659-5p	3.53E-06	0.12311587
hsa-miR-1322	3.70E-06	0.088120628
hsa-miR-551a	3.79E-06	0.214832043
hsa-miR-4313	3.79E-06	-0.421947447
hsa-miR-3126-5p	3.81E-06	0.205437325
hsa-miR-4651	3.85E-06	0.382555937
hsa-miR-3130-5p	4.08E-06	-0.151531633
hsa-miR-526a	4.23E-06	-0.226777462
hsa-miR-615-5p	4.29E-06	-0.232625142
hsa-miR-4417	4.29E-06	0.279602743
hsa-miR-934	4.29E-06	0.371360298
hsa-miR-154-5p	4.35E-06	-0.124820006
hsa-miR-632	4.37E-06	0.085885406
hsa-miR-1287	4.37E-06	0.136323138
hsa-miR-206	4.37E-06	0.140728581
hsa-miR-4731-3p	4.37E-06	-0.449517326
hsa-miR-633	4.46E-06	-0.137291153
hsa-miR-3129-3p	4.53E-06	-0.129875804
hsa-miR-628-3p	4.71E-06	0.136487461
hsa-miR-1299	4.75E-06	0.313028925
hsa-miR-584-3p	4.78E-06	0.112168624
hsa-miR-3144-5p	4.79E-06	0.162430441
hsa-miR-4284	4.79E-06	-0.448196268
hsa-miR-4768-3p	4.88E-06	0.125436797
hsa-miR-4703-5p	5.47E-06	-0.148813485
hsa-miR-5193	5.52E-06	0.171690429
hsa-miR-1268a	5.62E-06	0.279638522

hsa-miR-3680-3p	5.85E-06	0.102466742
hsa-miR-4659a-3p	5.88E-06	0.136222164
hsa-miR-3174	6.34E-06	0.321771855
hsa-miR-3622a-3p	6.96E-06	-0.09788673
hsa-miR-4754	7.12E-06	0.107941313
hsa-miR-3182	7.34E-06	-0.13461634
hsa-miR-2116-5p	7.45E-06	-0.119304614
hsa-miR-5683	7.69E-06	-0.119411894
hsa-miR-4281	7.75E-06	0.310080347
hsa-miR-597	7.94E-06	0.1428639
hsa-miR-26a-2-3p	7.94E-06	-0.176565203
hsa-miR-4428	8.19E-06	0.513302432
hsa-miR-517-5p	8.47E-06	-0.10265993
hsa-miR-299-3p	8.69E-06	-0.131392947
hsa-miR-296-3p	8.69E-06	-0.217253928
hsa-miR-3187-5p	9.14E-06	0.165013528
hsa-miR-3606	9.36E-06	-0.096463988
hsa-miR-519b-5p	9.50E-06	-0.235310338
hsa-miR-5088	9.91E-06	0.166844353
hsa-miR-4715-3p	9.99E-06	-0.136474208
hsa-miR-431-3p	1.03E-05	0.246509629
hsa-miR-1304-3p	1.03E-05	-0.540362054
hsa-miR-3664-3p	1.05E-05	0.100958282
hsa-miR-518d-3p	1.05E-05	-0.188017544
hsa-miR-3663-5p	1.12E-05	0.126281602
hsa-miR-636	1.12E-05	-0.240310464
hsa-miR-5001-5p	1.12E-05	0.2692002
hsa-miR-601	1.12E-05	0.28461213
hsa-miR-3190-3p	1.12E-05	0.330285052
hsa-miR-1321	1.19E-05	0.240985042
hsa-miR-4641	1.22E-05	-0.105772208
hsa-miR-518f-3p	1.24E-05	-0.138244076
hsa-miR-223-3p	1.24E-05	-0.399265312
hsa-miR-1267	1.25E-05	-0.432121002
hsa-miR-5590-5p	1.26E-05	-0.116909304
hsa-miR-140-3p	1.31E-05	-0.211956683
hsa-miR-4662a-5p	1.44E-05	-0.101526117
hsa-let-7i-5p	1.44E-05	-0.414069687
hsa-miR-548v	1.45E-05	-0.103894235
hsa-miR-1293	1.45E-05	0.195986369
hsa-miR-589-5p	1.52E-05	0.130252884
hsa-miR-3141	1.57E-05	0.299108162
hsa-miR-4639-3p	1.58E-05	0.151391271
hsa-miR-26b-5p	1.63E-05	-0.275425683
hsa-miR-4503	1.64E-05	0.096692869

hsa-miR-3915	1.66E-05	-0.252487111
hsa-miR-3121-5p	1.67E-05	-0.289018675
hsa-miR-4999-5p	1.70E-05	0.386389532
hsa-miR-4673	1.73E-05	0.204638534
hsa-miR-4652-3p	1.73E-05	-0.372782933
hsa-miR-4695-5p	1.75E-05	0.262797017
hsa-miR-4303	1.76E-05	0.162039877
hsa-miR-492	1.79E-05	0.167125549
hsa-miR-3115	1.81E-05	-0.10457127
hsa-miR-4767	1.81E-05	-0.146554122
hsa-miR-4270	1.81E-05	0.326144959
hsa-miR-4310	1.81E-05	-0.333294895
hsa-miR-1537	1.82E-05	-0.100848282
hsa-miR-106b-3p	1.91E-05	-0.13335186
hsa-miR-4482-5p	1.93E-05	0.115726055
hsa-miR-3975	1.95E-05	0.132082462
hsa-miR-3668	1.98E-05	-0.097450879
hsa-miR-3682-5p	2.00E-05	-0.107830677
hsa-miR-1306-5p	2.00E-05	-0.317000945
hsa-miR-297	2.07E-05	-0.122300357
hsa-miR-1273e	2.19E-05	0.374496386
hsa-miR-2681-3p	2.20E-05	-0.127590541
hsa-miR-361-3p	2.20E-05	-0.315736677
hsa-miR-4640-3p	2.20E-05	-0.360666667
hsa-miR-1915-3p	2.23E-05	0.284018567
hsa-miR-1253	2.30E-05	0.084046489
hsa-miR-4510	2.33E-05	-0.145350476
hsa-miR-4725-5p	2.33E-05	-0.213710878
hsa-miR-4676-5p	2.43E-05	0.099229864
hsa-miR-4259	2.51E-05	0.145431897
hsa-miR-378g	2.52E-05	0.09429993
hsa-miR-103a-2-5p	2.80E-05	0.086641627
hsa-miR-371b-5p	2.80E-05	0.348965979
hsa-miR-1911-5p	2.99E-05	-0.083054394
hsa-miR-5586-5p	2.99E-05	-0.099360565
hsa-miR-4777-5p	2.99E-05	-0.110780954
hsa-miR-487b	2.99E-05	0.156341336
hsa-miR-4488	2.99E-05	-0.36022786
hsa-miR-3193	3.07E-05	0.123812503
hsa-miR-3942-5p	3.09E-05	-0.088259032
hsa-miR-1255b-2-3p	3.09E-05	-0.119949502
hsa-miR-1225-5p	3.10E-05	0.324981295
hsa-miR-627	3.12E-05	0.140059923
hsa-miR-199a-5p	3.31E-05	-0.300686275
hsa-miR-33a-3p	3.56E-05	-0.128239665

hsa-miR-877-3p	3.70E-05	-0.16151426
hsa-miR-4271	3.73E-05	0.288318143
hsa-miR-3661	3.75E-05	0.119743402
hsa-miR-4498	3.83E-05	0.418591243
hsa-let-7b-3p	3.90E-05	-0.259092842
hsa-miR-2467-5p	4.13E-05	-0.119800612
hsa-miR-29a-3p	4.23E-05	-0.472916758
hsa-miR-4747-3p	4.61E-05	-0.259633737
hsa-miR-451a	4.67E-05	-0.614871769
hsa-miR-4532	4.74E-05	-0.216719593
hsa-miR-548c-3p	4.89E-05	-0.131214664
hsa-miR-1271-3p	4.92E-05	0.100535645
hsa-miR-202-5p	5.06E-05	-0.114361248
hsa-miR-4451	5.29E-05	0.193879165
hsa-miR-583	5.29E-05	-0.227382989
hsa-miR-5692a	5.36E-05	-0.12298318
hsa-miR-186-5p	5.55E-05	-0.078590473
hsa-miR-4639-5p	5.74E-05	0.272996015
hsa-miR-5586-3p	5.78E-05	-0.065428125
hsa-miR-150-5p	5.86E-05	-0.197473854
hsa-miR-3923	5.96E-05	0.083362306
hsa-miR-3681-5p	6.00E-05	0.080473518
hsa-miR-22-3p	6.01E-05	-0.464030232
hsa-miR-671-5p	6.15E-05	-0.308963725
hsa-miR-1269a	6.29E-05	-0.101148928
hsa-miR-449b-5p	6.49E-05	-0.104673154
hsa-miR-3154	6.54E-05	0.265089595
hsa-miR-3675-3p	6.85E-05	-0.360014526
hsa-miR-3156-5p	6.89E-05	0.291325048
hsa-miR-608	6.95E-05	-0.142690783
hsa-miR-2278	7.22E-05	-0.095672887
hsa-miR-603	7.43E-05	-0.088455172
hsa-miR-3183	7.45E-05	-0.068437177
hsa-miR-4796-5p	7.58E-05	0.120011321
hsa-miR-371b-3p	7.60E-05	-0.430976855
hsa-miR-150-3p	7.73E-05	0.148852278
hsa-miR-514a-5p	7.79E-05	-0.080191487
hsa-miR-1271-5p	7.79E-05	0.102057441
hsa-miR-23c	7.79E-05	-0.333043137
hsa-miR-3943	7.94E-05	-0.30856233
hsa-miR-4772-3p	8.27E-05	-0.146081221
hsa-miR-615-3p	8.32E-05	-0.264757784
hsa-miR-644b-3p	8.43E-05	0.168367633
hsa-miR-4492	8.50E-05	-0.219875089
hsa-miR-4472	8.72E-05	-0.292769646

hsa-miR-338-5p	8.84E-05	0.110912564
hsa-miR-5571-5p	8.89E-05	-0.34901175
hsa-miR-4436b-3p	8.98E-05	0.218379555
hsa-miR-3652	9.41E-05	0.258458668
hsa-miR-3177-5p	9.63E-05	0.082444415
hsa-miR-4289	9.78E-05	0.079808312
hsa-miR-3155a	9.82E-05	0.106530626
hsa-miR-3545-5p	9.87E-05	-0.082314082
hsa-miR-4253	9.96E-05	0.152067916
